# Myopic reallocation of extraction improves collective outcomes in networked common-pool resource games

**DOI:** 10.1038/s41598-020-79514-5

**Published:** 2021-01-13

**Authors:** Andrew Schauf, Poong Oh

**Affiliations:** Wee Kim Wee School of Communication and Information, Nanyang Technological University, Singapore, 639798 Singapore

**Keywords:** Complex networks, Ecological modelling, Ecological networks, Socioeconomic scenarios

## Abstract

When individuals extract benefits from multiple resources, the decision they face is twofold: besides choosing how much total effort to exert for extraction, they must also decide how to allocate this effort. We focus on the allocation aspect of this choice in an iterated game played on bipartite networks of agents and common-pool resources (CPRs) that degrade linearly in quality as extraction increases. When CPR users attempt to reallocate their extraction efforts among resources to maximize their own payoffs in the very next round (that is, myopically), collective wealth is increased. Using a heterogeneous mean-field approach, we estimate how these reallocations affect the payoffs of CPR users of different degrees within networks having different levels of degree heterogeneity. Focusing specifically on Nash equilibrium initial conditions, which represent the patterns of over-exploitation that result from rational extraction, we find that networks with greater heterogeneity among CPR degrees show greater improvements over equilibrium due to reallocation. When the marginal utility of extraction diminishes, these reallocations also reduce wealth inequality. These findings emphasize that CPR users’ adaptive reallocations of effort—a behavior that previously-studied network evolutionary game models typically disallow by construction—can serve to direct individuals’ self-interest toward the collective good.

## Introduction

A resource that is freely available to a community of users, but which provides reduced benefits as usage increases, is called a *common-pool resource* (CPR)^1–5^. Common-pool resources differ from *public goods*, which are *non-rivalrous*, meaning that increased usage by one individual does not reduce the benefits that a public good provides to other users. While various mechanisms underlying the collective maintenance of public goods in networked populations have been thoroughly investigated^6–8^, those of common-pool resources have yet to be as extensively studied using the combined tools of network science and evolutionary game theory. An ability to sustainably make use of degradable natural resources—for example fisheries, pastures, forests, or water resources—is obviously crucial to human survival. In addition to these more tangible resources, though, human activities are also increasingly reliant upon resources that facilitate communication and the collective sharing of information; because many of these resources may suffer reduced performance if subjected to overuse^9–11^, their effective management may also benefit from a deeper understanding of the dilemmas faced by CPR users within complex, structured populations. Research into CPR management often stresses that there is no panacea for comprehensively framing and understanding all CPR dilemmas^12^. Nonetheless, network models provide a promising framework within which to study how the basic mechanisms involved in the use of rivalrous resources play out on complex structured populations, and so to help bridge the gap between the more abstract insights of network evolutionary game theory and the special-case issues faced in real-world CPR management.

Perhaps the most salient overarching theme of the study of CPRs has been that users often regulate their own usage in ways that avert resource degradation more effectively than can any regulation imposed by a centralized decision-maker^3^. Information sharing, cultural norms, and the negotiation of various ad hoc rules have all been observed to facilitate this self-regulation in real-world CPR systems^3,13,14^. But the observation of these common features begs a more fundamental question: how might these rules and norms have evolved in the first place? Evolutionary game theory provides a useful framework to study various mechanisms by which social individuals—despite being initially motivated only by short-sighted self-interest—may come to coordinate their actions in ways which avert the “tragedy of the commons” and benefit the collective good. In particular, network models have been used to show that the structural properties of social networks can affect the extent to which cooperation can persist and spread within a population. This idea has most often been demonstrated within the context of network *public goods game* (PGG) models^6–8^. In these models, degree heterogeneity among agents (i.e. the numbers of distinct public goods among which agents diversify their investments) and group sizes (i.e. the numbers of users of each public good) have each been shown to play a role in shaping the prevalance of cooperation when strategies evolve under imitation, that is, when individuals adopt the strategies of their higher-fitness neighbors. However, since these network models have typically dealt with public goods, they may fail to capture some fundamental aspects of the unique dilemmas faced by users of rivalrous common-pool resources. A variety of agent-based models have been developed to address more special-case CPR dilemmas^15–20^ and to investigate the evolution of rules governing CPR usage on lattice-structured populations^21–24^ or networks^25^. As in the aforementioned network PGG models, most of these CPR models presume that a specific, pre-defined cooperation rule is under consideration, which is designed to yield longer-term benefits if widely adopted. Some additional social mechanism may then prompt short-sighted individuals to voluntarily adopt this proposed rule despite the more immediate advantages that stand to be gained by defecting from it.

Fewer studies have examined the relationship of population structure to CPR self-regulation at a more fundamental level, however. İlkılıç considered a CPR extraction game in which a population of agents exert effort to extract benefits from multiple sources, and where agents’ access to these sources are described by bipartite networks^26^. In that model, CPR sources were assumed to degrade linearly with respect to the total extraction to which they were subjected. Agents’ payoff functions also included a quadratic cost term to incorporate diminishing marginal utility. This work detailed methods for computing the extraction levels in the game’s unique Nash equilibrium state, wherein individuals seek to maximize their own *individual* payoffs, and Pareto efficient states, in which a population’s *collective* wealth is maximized, for any given bipartite network of agents and sources. These methods were then demonstrated on example networks having only a few nodes. In this article, we elaborate upon İlkılıç’s model, aiming not only to compute certain extraction states of interest for specific networks, but rather to understand the *patterns* of over-exploitation and inequality that emerge in these states, and how they are shaped by population structure. More specifically, we wish to clarify how resource over-exploitation or agent payoffs depend on a node’s network degree, and how the degree distributions of the surrounding network determine this dependence.

While previous network PGG models typically presume that agents apply a single pre-defined strategy (i.e. either “cooperation” or “defection”) in a uniform way to all of their affiliated goods, this model allows agents to selectively allocate their efforts among multiple CPRs. We investigate the role of this allocation decision by introducing a **CPR reallocation game** in which agents adaptively shift their extraction efforts between CPRs, updating their *allocations* of effort without varying the overall *magnitudes* of effort that they exert. This update rule is **myopic** in the sense that it aims only to increase an agent’s payoffs in the next iteration of the game based on the resource conditions observed in the current round. Unlike the rational behavior that leads to Nash equilbrium, this reallocation update rule models extraction dynamics when agents’ rationality is bounded by the restriction that agents do not vary their overall extraction levels over time, but rather view CPR extraction purely as an allocation problem. This premise allows us to explore the extent to which the “tragedy of the commons” can be alleviated by reallocations alone, moving the population closer to optimal states even in the absence of regulation by any centralized decision-maker.

We demonstrate that when network-structured populations overuse CPRs, these myopic reallocations will improve the population’s collective wealth, thus partially averting the “tragedy of the commons”. If individuals all unilaterally attempt to maximize their own payoffs, as in Nash equilibrium, then the extent to which each CPR is over-exploited depends on its number of users (i.e. its degree). A network’s degree distributions thus determine how inefficiently the system operates under this rational extraction behavior, and so also determine how much a population potentially stands to gain from reallocation by comparing Nash equilibria with the **steady states** that the system subsequently approaches under myopic reallocation dynamics. To estimate how these gains are distributed throughout a population, we use a *heterogeneous mean-field* perspective, which bins a network’s nodes by degree and estimates the mean properties of each degree class using degree distributions^27^. We show that in the absence of diminishing marginal utility, these gains tend to be distributed among agents of all degree classes in proportion to their extraction efforts, so that these collective gains are not necessarily at odds with any individual’s self-interest. However, when diminishing marginal utility acts to limit individuals’ extraction levels at equilibrium, these reallocations also tend to increase the equality of the resulting payoff distributions by partially levelling out degree-based payoff inequities that emerge under rational extraction. Our results emphasize that users’ decisions regarding their allocations of effort among multiple degradable resources—an aspect of agent choice which is typically excluded by construction from more widely-studied network PGG models, or even previous network CPR models—can potentially drive populations of self-interested individuals toward more collectively optimal states in ways that are shaped by network topology.

## Methods

### Agent-resource affiliation networks

We consider games played on bipartite networks of $M$ agents and $N$ sources in which the presence of an edge between an agent and a source indicates that the agent has access to that source. We assume that this network structure is predetermined exogenously, and remains static over the time scales of interest. The set of all sources is denoted as $\mathbf{S}$, while the set of sources affiliated with a particular agent $a$ is denoted as ${\mathbf{S}}_{a}$ and the agent’s degree (i.e. its number of affiliated sources) as $m(a)$. We denote the set of all agents as $\mathbf{A}$, the set of agents affiliated with a particular source $s$ as ${\mathbf{A}}_{s}$, and this source’s degree (i.e. its number of affiliated agents) as $n(s)$. Mean values for quantities over the set of sources are denoted with brackets: $\langle x\rangle =\left[{\sum }_{s\in \mathbf{S}}x(s)\right]/N$. The distribution of agent degrees within the population is denoted as ${P}_{\mathbf{A}}(m)$, and the distribution of source degrees is denoted as ${P}_{\mathbf{S}}(n)$.

**Figure 1:**
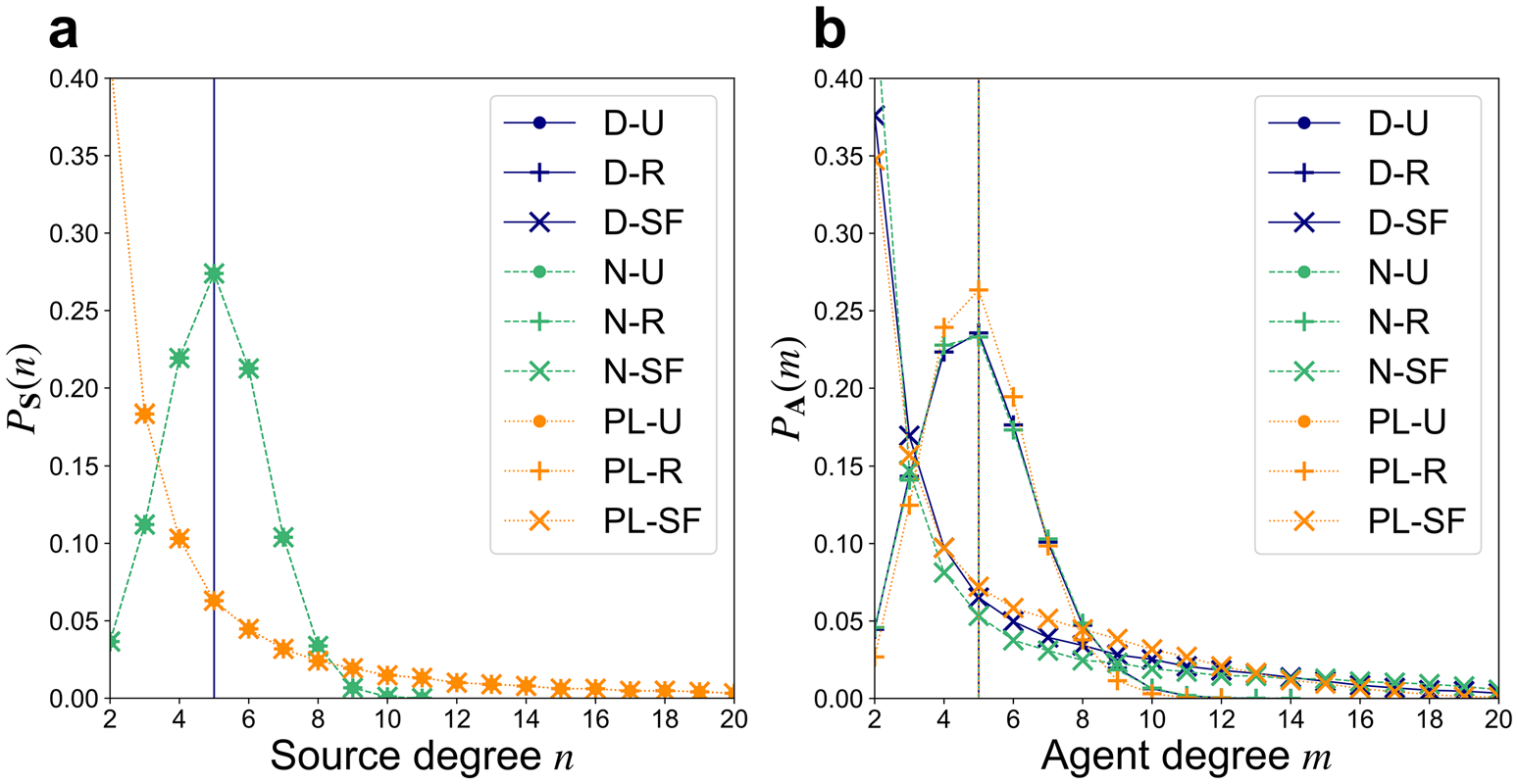
(**a**) CPR source degree distributions, and (**b**) agent degree distributions from 9 network ensembles, each representing a combination of a delta-function (D), normal (N), or power-law (PL) source degree distribution with a uniform (U), random (R), or scale-free-type (SF) agent degree distribution. Source degree distributions are identical for each of the 3 ensembles that share a common source degree distribution type, hence the overlapping curves.

Results presented below are based on ensembles of $1{0}^{3}$ network realizations, each of which have $M=50$ agents, $N=50$ sources, and share mean agent degree $\langle m\rangle =5$ and mean source degree $\langle n\rangle =5$. We consider 9 network ensembles, each generated to have a particular combination of one of three source degree heterogeneity types—delta-function (“D”), normal (“N”), or power-law (“PL”)—with one of three agent degree heterogeneity types—uniform (“U”), random (“R”), or scale-free-type^28^ (“SF”) (see Section S1.1 of the Supplementary Information). We denote the ensemble of networks having normal (“N”) source degree distributions and random (“R”) agent degree distributions as “N-R”, for example. From each ensemble, we extract degree histograms to represent the distributions ${P}_{\mathbf{A}}(m)$ and ${P}_{\mathbf{S}}(n)$ (Fig. 1).

### Common-pool resource extraction game

Following and generalizing upon the approach of İlkılıç^26^, we consider the extraction game in which each agent $a$ exerts an extraction effort of magnitude $q(a,s)$ toward extraction from an affiliated source $s$; an agent’s strategy thus comprises its choices of $q(a,s)$ for each source from its set of affiliated sources ${\mathbf{S}}_{a}$. We denote the total extraction effort exerted by an agent as $\overleftarrow{q}(a)$, and the total extraction effort exerted upon a source by $\overrightarrow{q}(s)$, which we will refer to as the **extraction pressure** on source $s$. We define the **quality**, $b(s)$, of a source $s$ as the magnitude of the benefit it returns to a user in return for each unit of extraction pressure exerted upon it. The total fitness of an agent $a$ is thus given by the *payoff function*

1 $$f(a)=\left[\sum_{s\in {\mathbf{S}}_{a}}q(a,s)\cdot b(s)\right]-\frac{\gamma }{2}\overleftarrow{q}(a{)}^{2} ,$$

where the constant coefficient $\gamma \ge 0$ of the quadratic cost term quantifies the relative influence of diminishing marginal utility^26^. Extraction is *non-excludable*, and so is not regulated by any centralized decision-maker, nor does it involve any negotiations or transactions between agents^1–3^; this cost term represents only the inherent cost of the act of extraction itself, which is effectively increased at higher levels of extraction by diminishing marginal utility. In common-pool resources, an additional effective cost of extraction, shared by all users of a given CPR, comes from the property that extraction by one user reduces the benefits available to others^1–3^. This rivalrous (or *subtractable*) property of CPRs is modelled here by assuming that each source offers users some basic benefit per unit extraction effort, $\alpha >0$, which is reduced by some amount $\beta (s)>0$ for each unit of total extraction pressure that the source receives, so that

2 $$b\left(s\right)=\alpha -\beta \left(s\right)\overrightarrow{q}\left(s\right).$$

The dependence of the parameter $\beta$ on source $s$ allows the model to capture variability among the characteristics of individual sources (the Supplementary Information further considers a case where $\alpha$ and $\gamma$ may also vary among nodes). Given our focus on the relationship of network degree to extraction, in the cases shown here we define $\beta$ as a function of source degree $n$ (as denoted by a subscript: ${\beta }_{n}$); we then follow previous network PGG literature^6^ by examining two “extreme cases”, each representing a distinct type of dependence of the parameter of interest upon degree. Here, the first of these is a ***uniform capacity scenario***, where all sources degrade in an identical manner, such that $\beta (s)\equiv \beta$ for some constant $\beta >0$. This corresponds to the game previously studied by İlkılıç^26^. In addition, we introduce a ***degree-proportional capacity scenario*** wherein $\beta (s)={\beta }_{0}\langle n\rangle /n(s)$ for some constant ${\beta }_{0}$, so that each source’s quality degrades in proportion to the mean extraction pressure *per user* that it receives. As we will show, the major results of this article are robust with respect to these changes in the assumptions made about the dependence of CPR degradation characteristics on degree.

### Reallocation dynamics

Within the context of the CPR extraction game described above, we consider the dynamics which occur if each agent $a$ is free to adaptively reallocate their extraction efforts among their multiple affiliated sources without altering the overall magnitudes $\overleftarrow{q}(a)$ of their extraction efforts. We assume that agents have access to accurate information about the current quality $b(s)$ of each of their affiliated sources $s$ at each iteration, and anticipate that extraction from currently higher-quality resources will lead to higher payoffs in the next round. Following each round of an iterated game, myopic agents thus compare the quality values of their affiliated sources, and attempt to maximize their payoffs in the subsequent round by shifting their efforts from lower- to higher-quality sources. Agents’ rates of performing these updates are assumed to be proportional to the increases in payoffs that they expect to achieve thereby, as in a discrete replicator rule^6^. That is, their rates of reallocation between any two sources are proportional to the differences in quality between these sources, so that each extraction level $q(a,s)$ evolves according to

3 $$\frac{\mathrm{d}}{\mathrm{d}t}q(a,s)=k\sum_{s{^{\prime}}\in {\mathbf{S}}_{a}}\left[b(s)-b(s{^{\prime}})\right],$$

where $k>0$ is some constant. These reallocation dynamics conserve each agent’s total extraction level $\overleftarrow{q}(a)$ in time, as the system approaches *steady states* in which all sources sharing some affiliated agents in common also share a common quality value. These steady states are distinct from the unique Nash equilibria of the CPR extraction game; furthermore, even within this CPR reallocation game where agents are restricted to only perform reallocation moves that conserve the total magnitudes of their extraction efforts, there typically do exist reallocation moves whereby an agent could increase its own fitness if all other agents held their extraction levels constant (see Section S2.2 of the Supplementary Information).

We use a heterogeneous mean-field approach to estimate the dependence of the *expected source extraction pressure* $\langle \overrightarrow{q}{\rangle }_{n}$ on source degree $n$, and the dependence of *expected agent payoff* $\langle f{\rangle }_{m}$ on agent degree $m$ for Nash equilibrium and Pareto efficient states (as detailed in Section S3 of the Supplementary Information). We then estimate the shifts in CPR extraction pressure and agent payoffs that are brought about as the dynamics of Eq. (3) bring Nash equilibrium initial conditions toward steady states. For specific network realizations, steady states of these reallocation dynamics can be computed as solutions of a linear complementarity problem (see Section S5.1 of the Supplementary Information). To validate these estimates, we also compute Nash equilibrium states, Pareto efficient states, and steady states of reallocation dynamics from equilibrium for each of the specific network realizations generated (as detailed in Sections S5 and S6 of the Supplemental Materials).

## Results

### Myopic reallocation improves collective wealth

Beginning from some initial extraction state, agents within a networked population of multiple common-pool resources play an iterated game in which they observe current resource conditions at each round, and incrementally shift their extraction efforts from lower-quality sources toward higher-quality sources in order to maximize their payoffs in the following round (Eq. 3). Agents’ extraction efforts are thus redirected away from over-exploited sources toward less-exploited sources so that the system approaches a steady state in which all sources equally share the burden of over-extraction. In the process, some sources increase in quality, while others are further degraded; nonetheless, the overall result of these reallocations is a net increase in collective wealth.

To show this, we consider an arbitrary initial extraction state, in which the population’s collective extraction effort is $Q=N\langle \overrightarrow{q}\rangle$. In this state, the initial collective payoff extracted by the population is ${F}_{0}={\sum }_{s\in \mathbf{S}}\overrightarrow{q}(s)\cdot b(s)$ (where we ignore cost terms, since these remain constant under reallocation), and so the population’s collective wealth per unit extraction effort is

4 $$\frac{{F}_{0}}{Q}=\frac{\sum_{s\in \mathbf{S}}\overrightarrow{q}(s)\cdot \left[\alpha -\beta (s)\overleftarrow{q}(s)\right]}{\sum_{s\in \mathbf{S}}\overrightarrow{q}(s)}=\alpha -\frac{\langle \beta {\overrightarrow{q}}^{2}\rangle }{\langle \overrightarrow{q}\rangle }.$$

Under reallocation dynamics (Eq. 3), this total extraction $Q$ is conserved, and the system will approach a steady state in which all sources share a common quality value

5 $${b}_{f}=\alpha -\frac{\langle \overrightarrow{q}\rangle }{\langle {\beta }^{-1}\rangle }.$$

The population’s collective wealth approaches the steady-state value

6 $${F}_{f}={\sum }_{s\in \mathbf{S}}\left[\overrightarrow{q}(s)\cdot {b}_{f}\right]=Q{b}_{f}.$$

Collective wealth is increased (or at least conserved) if ${F}_{0}\le {F}_{f}$, or equivalently, if $\frac{{F}_{0}}{Q}\le {b}_{f}$. Using Eqs. 4 and 5, this condition reduces to

7 $$\langle \overrightarrow{q}{\rangle }^{2}\le \langle \beta {\overleftarrow{q}}^{2}\rangle \langle {\beta }^{-1}\rangle .$$

The validity of this inequality is guaranteed by the Cauchy–Schwarz inequality^29^, $\langle XY{\rangle }^{2}\le \langle {X}^{2}\rangle \langle {Y}^{2}\rangle$ for random variables $X$ and $Y$, with the identifications $X=\sqrt{\beta (s)}\overrightarrow{q}(s)$ and $Y=\sqrt{\beta (s{)}^{-1}}$. Furthermore, equality occurs if and only if the quantity $\beta \left(s\right)\overrightarrow{q}\left(s\right)$ shares the same value for all sources, that is, when initial conditions are already steady states where all sources share a common quality value. Reallocation dynamics thus increase collective wealth for any initial condition where sources vary from one another in quality (see Section S2.1 of the Supplementary Information). This includes Nash equilibrium initial conditions, upon which we will now focus our attention.

### CPR degree heterogeneity leads to greater improvements in efficiency under myopic reallocation

In the unique Nash equilibrium state of a given network^26^, each agent sets its extraction at each source to the point beyond which further extraction would increase its costs more than it would increase its payoffs, given that all other agents are doing the same. In this state, no agent can increase its payoffs by *unilaterally* adjusting its extraction levels while other agents hold their extraction levels constant. However, when all agents *simultaneously* adapt their extraction levels according to the reallocation update rule (Eq. 3), under which each increase in extraction at one source is matched by an equal decrease at another source, then higher payoffs can be achieved. To quantify the extent to which reallocation alone can help alleviate the “tragedy of the commons” represented by Nash equilibrium, we now apply reallocation dynamics to Nash equilibrium initial conditions on a variety of network types, and compare the population’s collective wealth values before and after reallocation.

When network-structured populations of rational individuals extract benefits from multiple linearly-degrading CPRs, the burdens of over-exploitation tend to fall upon sources in a degree-dependent manner. Myopic reallocation tends to shift these burdens among sources of different degrees, and to distribute the resulting increases in collective wealth among individuals of different degree classes. In order to understand how these reallocations shift extraction pressure and agent payoffs among nodes of different degrees, we use a heterogeneous mean-field approach to derive estimates for these shifts. Under this perspective, the conditions defining Nash equilibrium ($\frac{\partial f(a)}{\partial q(a,s)}=0$) lead us to estimate the expected values for extraction pressure on degree-$n$ sources, $\langle \overrightarrow{q}{\rangle }_{n}$, by solving a linear system defined by

8 $$\langle \overrightarrow{q}{\rangle }_{n}=\frac{1}{{\beta }_{n}}\left[\frac{n}{n+1}\right]\left[\alpha -\sum_{m=1}^{{m}_{\mathrm{max}}}{P}_{\mathbf{A}}\left(m\right)\frac{m}{\langle m\rangle }\cdot \left(\frac{\gamma m}{[\gamma m\langle {\beta }^{-1}{\rangle }_{m}+1]}\left[\alpha \langle {\beta }^{-1}{\rangle }_{m}-\sum_{{n}^{^{\prime}}=1}^{{n}_{\mathrm{max}}}{P}_{\mathbf{S}}({n}^{^{\prime}})\frac{{n}^{^{\prime}}}{\langle n\rangle }\cdot \langle \overrightarrow{q}{\rangle }_{{n}^{^{\prime}}}\right]\right)\right],$$

with one such condition for each unique source degree $n\in \{1,\dots , {n}_{\mathrm{max}}\}$ represented in the network, where brackets subscripted by agent degree $m$ indicate expected values $\langle x{\rangle }_{m}={\sum }_{n=1}^{{n}_{\mathrm{max}}}{P}_{\mathbf{S}}\left(n\right)\frac{n}{\langle n\rangle }\cdot {x}_{n}$ and we have assumed no degree-degree correlations (see the Supplementary Information Section S3 for details). Solving this system numerically (here we use Python 3.7.3 with SciPy 1.2.1^30^) for each of the 9 network types under consideration by inserting the corresponding ensemble degree distributions ${P}_{\mathbf{A}}\left(m\right)$ and ${P}_{\mathbf{S}}\left(n\right)$ (Fig. 1), we use the resulting values of $\langle \overrightarrow{q}{\rangle }_{n}$ to compute the expected total extraction by a degree-*m* agent $\langle \overleftarrow{q}{\rangle }_{m}$ at equilbrium as

9 $$\langle \overleftarrow{q}{\rangle }_{m}=\left(\frac{m}{m\gamma \langle {\beta }^{-1}{\rangle }_{m}+1}\right)\left[\alpha \langle {\beta }^{-1}{\rangle }_{m}-\left(\sum_{n=1}^{{n}_{\mathrm{max}}}{P}_{\mathbf{S}}\left(n\right)\frac{n}{\langle n\rangle }\cdot \langle \overrightarrow{q}{\rangle }_{n}\right)\right],$$

from which $\langle q{\rangle }_{m,n}$, the expected equilibrium extraction by a degree-$m$ agent from a degree-$n$ source, can be computed using the Nash equilbrium condition:

10 $$\langle q{\rangle }_{m,n}=\frac{\alpha }{{\beta }_{n}}-\langle \overrightarrow{q}{\rangle }_{n}-\frac{\upgamma }{{\beta }_{n}}\langle \overleftarrow{q}{\rangle }_{m}.$$

**Figure 2:**
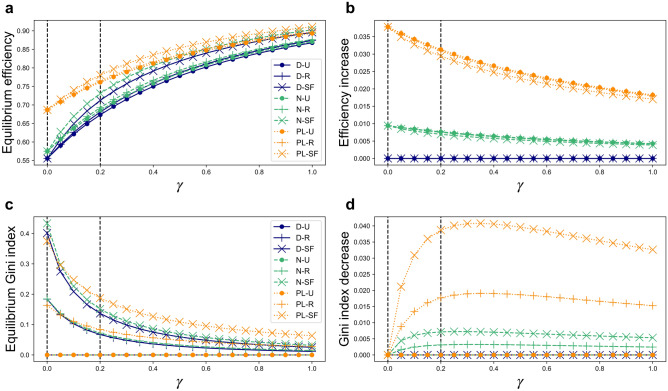
Estimates of (**a**) Ratio of total collective wealth of equilibrium (“Eq”) states relative to efficient (“Ef”) states, ${F}_{\mathrm{Eq}}/{F}_{\mathrm{Ef}}$; (**b**) increase in efficiency from equilibrium to steady states (“SS”), $({F}_{\mathrm{SS}}-{F}_{\mathrm{Eq}})/{F}_{\mathrm{Ef}}$; (**c**) Gini index of equilibrium states ${G}_{\mathrm{Eq}}$; and (**d**) decrease in Gini index from equilibrium to steady states, $({G}_{\mathrm{Eq}}-{G}_{\mathrm{SS}})$, all as functions of cost parameter $\gamma$. Results shown correspond to a *uniform capacity* scenario with $\alpha =\beta =1$.

**Figure 3:**
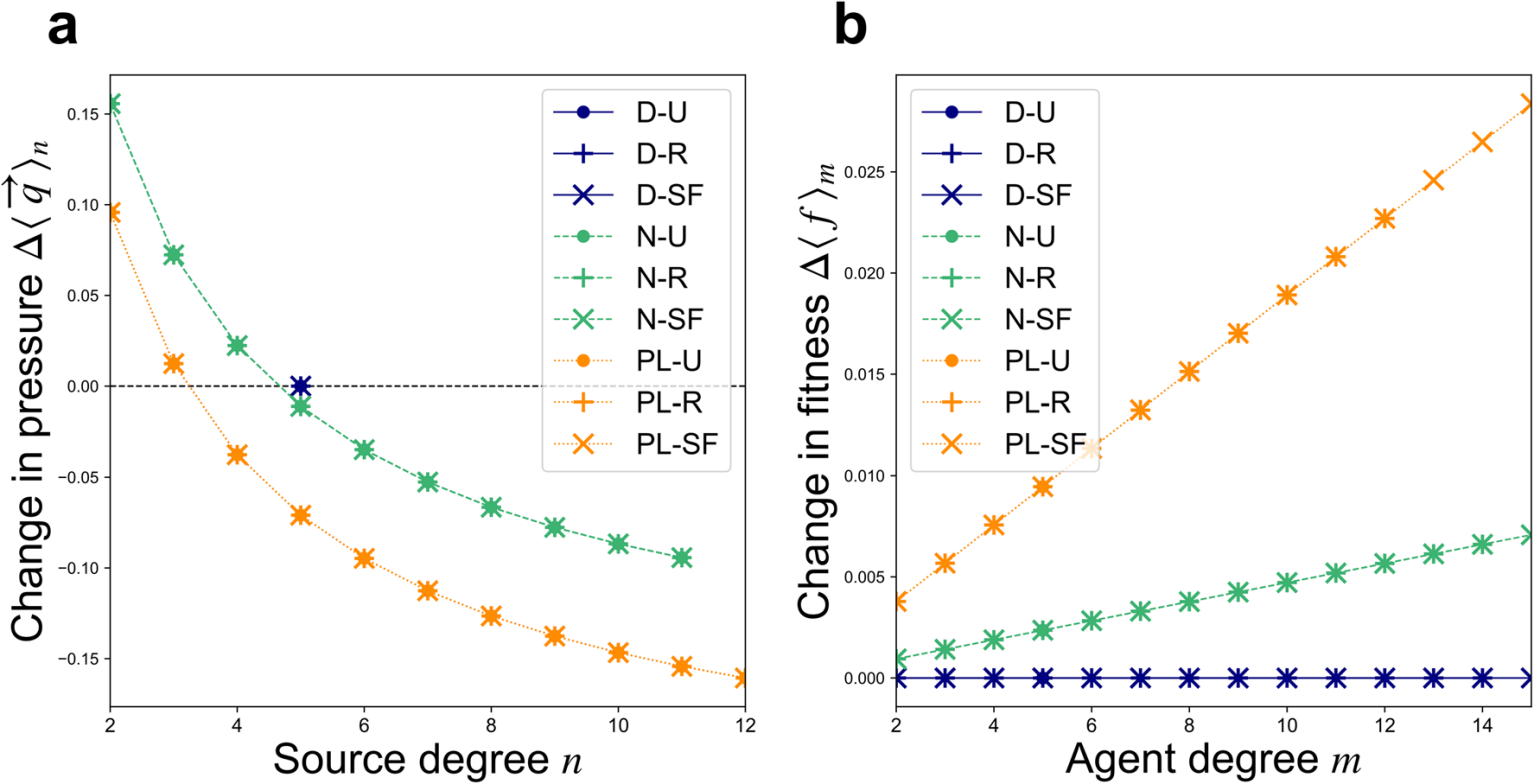
Estimated shifts in extraction patterns due to reallocation dynamics from Nash equilibrium (“Eq”) to steady states (“SS”) under cost-free extraction: (**a**) Change in total extraction pressure $\Delta \langle \overrightarrow{q}{\rangle }_{n}=\langle \overrightarrow{q}{\rangle }_{n,\mathrm{SS}}-\langle \overrightarrow{q}{\rangle }_{n,\mathrm{Eq}}$, as a function of source degree $n$; and (**b**) change in expected agent fitness, $\Delta \langle f{\rangle }_{m}=\langle f{\rangle }_{m,\mathrm{SS}}-\langle f{\rangle }_{m,\mathrm{Eq}}$ as a function of agent degree $m$. Results shown correspond to a *uniform capacity* scenario with $\alpha =\beta =1$ and $\gamma =0$. Note that results for all network types sharing a common source degree distribution type (“D”, “N”, or “PL”) are overlapping.

**Figure 4:**
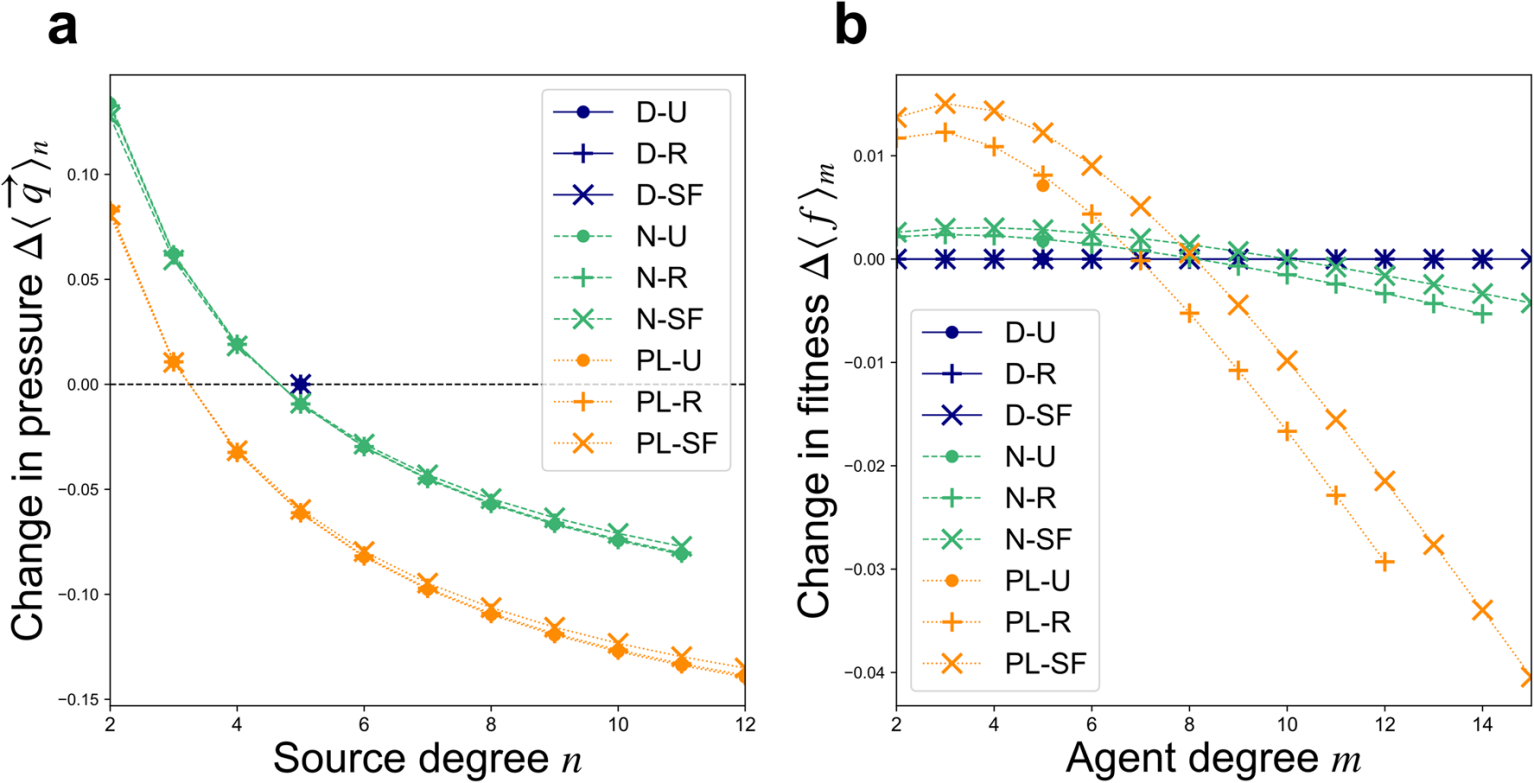
Estimated shifts in extraction patterns due to reallocation dynamics from Nash equilibrium (“Eq”) to steady states (“SS”) under costly extraction: (**a**) Change in total extraction pressure $\Delta \langle \overrightarrow{q}{\rangle }_{n}=\langle \overrightarrow{q}{\rangle }_{n,\mathrm{SS}}-\langle \overrightarrow{q}{\rangle }_{n,\mathrm{Eq}}$, as a function of source degree $n$; and (**b**) change in expected agent fitness, $\Delta \langle f{\rangle }_{m}=\langle f{\rangle }_{m,\mathrm{SS}}-\langle f{\rangle }_{m,\mathrm{Eq}}$ as a function of agent degree $m$. Results shown correspond to a *uniform capacity scenario* with $\alpha =\beta =1$ and $\gamma =0.2$.

These values are then used to compute the corresponding estimated collective wealth (i.e. the sum of all agent payoffs, $F=\sum_{a\in \mathbf{A}}f(a)$) and wealth equality (as quantified by Gini index $G$) attained at Nash equilibrium, as well as the subsequent shifts that are brought by myopic reallocation dynamics toward steady states. These values are shown in Fig. 2 for a range of values of the cost parameter $\gamma$, which quantifies the influence of diminishing marginal utility. The expected changes in extraction pressure for sources of different degrees, as well as the changes in agent fitness expected for agents of each degree class, are illustrated for each network type for cost-free extraction ($\gamma =0$) in Fig. 3, and similarly for a representative case of costly extraction ($\gamma =0.2$) in Fig. 4. The estimates presented here correspond to a *uniform capacity* scenario where all CPRs degrade in proportion to the total amount of extraction exerted upon their users. However, we find that qualitatively similar results also hold for a *degree-proportional capacity* scenario in which sources degrade in proportion to the total extraction *per user* that they receive (see Section S4 in the Supplementary Information).

In Nash equilibrium states of the *uniform capacity* scenario, sources with fewer users (i.e. lower degree) experience lower extraction pressure. Since all networks under comparison here share an equal number of edges, networks having greater heterogeneity among source degrees—and thus a greater abundance of low-degree sources—suffer less over-exploitation overall, and so tend to operate more efficiently at equilibrium (Fig. 2). As agents then shift their extraction away from over-burdened, lower-quality sources toward higher-quality sources, these systems approach steady states where their multiple CPR sources all share a uniform quality value. In this way, steady states of reallocation dynamics qualitatively resemble Pareto efficient extraction states, which are characterized by uniform quality among all CPR sources (though, unlike these steady states, optimal efficiency also requires uniform extraction levels among all agents regardless of degree; see Section S3.2 in the Supplementary Information). The resulting shifts in efficiency (Fig. 2b), source extraction pressure (Figs. 3a and 4a), and agent payoffs (Figs. 3b and 4b) are more pronounced for networks having greater heterogeneity among CPR source degrees due to the greater initial discrepancies among source quality values that these networks support at Nash equilibrium. When simulations of reallocation dynamics from equilbrium are performed on individual networks (see Section S6 in the Supplementary Information), then the shifts in extraction pressure and agent payoffs observed are often more exaggerated than those estimated here. Since the heterogeneous mean-field perspective treats all sources of a common degree as a single class, it does not distinguish higher-order differences among nodes that share the same degree. As a result, the model predicts *no* shifts under reallocation dynamics for networks in which all sources share a common degree, i.e. delta-function (“D”) source degree distributions, for example. However, on actual networks of this type, reallocation dynamics nonetheless *do* increase collective wealth by equalizing differences in quality among sources.

When extraction is costly ($\gamma >0$), agent degree heterogeneity also plays a secondary role to source degree heterogeneity in determining equilibrium efficiency and the effects of reallocation dynamics (Figs. 2 and 4). Diminishing marginal utility motivates agents to moderate their overall extraction levels; all sources affiliated with any given agent will be affected by its tendency to reduce extraction, and the extent of this reduction will depend in turn on each source’s degree, the degrees of its other users, and so on. Higher agent degree heterogeneity is thus predicted to slightly increase equilibrium efficiency due to the presence of higher-degree agents that reduce their extraction per source by larger amounts than do lower-degree agents. While the overall gains in collective wealth expected to be achieved by way of reallocations are thus slightly reduced by the presence of these higher-degree agents, greater agent degree heterogeneity is also associated with *faster times of convergence* toward steady states, since high-degree agents are able to simultaneously shift efforts directly between a large number of sources, and so to more rapidly equalize source quality values (see Section S5.1 in the Supplementary Information).

### Myopic reallocation from Nash equilibrium reduces wealth inequality

Since reallocation dynamics increase collective wealth, many—if not all—agents will attain improved payoffs under reallocation dynamics from suboptimal states like Nash equilibrium. We now turn our attention to *how* these increases in collective wealth are distributed throughout a population with respect to agent degree. Under the heterogeneous mean-field approach, we estimate that the shift in expected payoffs due to reallocations from Nash equilibrium are given by

11 $$\Delta \langle f{\rangle }_{m}=m\left[\left(\frac{1}{\langle n\rangle }\left[\langle \frac{n{b}_{n}}{{\beta }_{n}}\rangle {b}_{f}-\langle \frac{n{b}_{n}^{2}}{{\beta }_{n}}\rangle \right]\right)-\upgamma \langle \overleftarrow{q}{\rangle }_{m}\left(\frac{1}{\langle n\rangle }\left[\langle \frac{n}{{\beta }_{n}}\rangle -\langle \frac{n{b}_{n}^{2}}{{\beta }_{n}}\rangle \right]\right)\right],$$

where ${b}_{n}=\alpha -{\beta }_{n}\langle \overrightarrow{q}{\rangle }_{n}$ (see Section S3.1.3 in the Supplementary Information). When extraction is cost-free ($\gamma =0$), the increased payoffs brought about by reallocation dynamics are expected to affect each edge in a uniform way, on average, and thus tend to be shared among agents of all degree classes in proportion to their degree $m$. This is reflected in the linear increase of expected agent payoff with respect to degree (Fig. 3b), and also in the lack of change in the expected Gini index predicted for all network types under cost-free ($\gamma =0$) extraction (Fig. 2d). However, when extraction is costly ($\gamma >0$) and diminishing marginal utility acts to disincentivize increased extraction for higher-degree agents, the overall efficiency (Fig. 2a) and equality (Fig. 2c) of equilibrium states are increased from those observed under cost-free extraction. In these cases, reallocation dynamics also tend to increase the *equality* of the population’s wealth distribution, as reflected in the decreasing—and eventually negative—shifts in payoffs expected for agents of increasingly high degree (Fig. 4b), and also in the expected reductions in Gini index (Fig. 2d), caused by reallocation dynamics. This occurs because diminishing marginal utility motivates high-degree agents to exert less overall extraction effort *per source* at Nash equilibrium than do lower-degree agents. In the steady states subsequently reached under reallocation dynamics, all sources share a uniform quality value; each agent’s total extracted benefits then becomes strictly proportional to the overall magnitude of its extraction effort. Higher-degree agents end up receiving a smaller payoff *per source* than do their lower-degree counterparts in steady states. As Eq. (11) suggests, agents with higher initial extraction levels $\langle \overleftarrow{q}{\rangle }_{m}$ will experience a lower (and possibly even negative) shift in payoff per source $\Delta \langle f{\rangle }_{m}/m$ as a result of reallocations. This levelling-out of degree-based payoff inequities has its most pronounced effects at intermediate levels of the cost parameter (here, for values of $\gamma \approx .35$, as shown in Fig. 2d). In simulations performed on specific networks, we find that reallocation dynamics lead not only to increased collective wealth, but also to increased *equality*, even on networks with homogeneous, “delta-function” (“D”) source degree distributions, although the heterogeneous mean-field approach predicts no such shift. Networks of other types similarly tend to undergo greater increases in equality than those predicted here due to higher-order types of heterogeneity not captured by the model (see Section S6 in the Supplementary Information).

## Discussion

We have shown that in an evolutionary game which models common-pool resource extraction by network-structured populations, individuals’ myopic reallocations of CPR usage among their multiple affiliated sources tend to improve collective wealth. We show that the “price of anarchy” (as quantified, for example, by the inefficiency of the outcomes reached under rational extraction, i.e. Nash equilibrium^31,32^) is primarily dependent on degree heterogeneity among the system’s multiple resources. This “price of anarchy” can be significantly reduced, however, if agents’ short-sighted self-interest is channeled through an allocation decision, as modelled by the reallocation update rule considered here, rather than through rational choice made in light of complete and perfect information under no such constraint. The extent of this improvement is greater in networks with higher heterogeneity among CPR degrees. In the case of cost-free extraction (or under a linear cost function; see Section S3.1.3 in the Supplementary Information), the improvements gained through these reallocation moves tend to be shared in an egalitarian way across all of a network’s edges. In the case that diminishing marginal utility acts to disincentivize high levels of extraction at Nash equilibrium, though, these reallocations tend to redistribute payoffs in ways that disproportionately benefit lower-degree agents, and so also increase the *equality* of the population’s wealth distribution. The results presented here emphasize the potential importance of these allocation-related decisions in guiding networked populations to overcome CPR dilemmas, and possibly even to achieve more *egalitarian* outcomes than those reached under rational extraction.

In the networks and conditions considered here, the improvements gained through these reallocations are relatively small with respect to a population’s overall wealth, and certainly smaller than the gains that could be achieved if agents were inclined to reduce the overall *magnitudes* of their extraction efforts. Nonetheless, this reallocation game provides an analogue to results from typical network evolutionary PGGs; in both cases, we find that the self-interest of myopic individuals can be directed toward outcomes which improve collective wealth to an extent that is determined by a network’s degree heterogeneity. The game studied here considers the how agents adapt their *allocations* while holding the overall *magnitudes* of their efforts fixed. This stands in contrast to typical PGG models, which assume that agents vary their strategies in *magnitude* while holding their *allocations* of effort fixed across all goods. Their conclusions rely heavily on this assumption; were individuals capable of selectively reallocating their contributions toward cooperation-dominated games, the network’s multiple PGGs would become decoupled, eliminating the very mechanism by which cooperative strategies can be introduced into initially defection-dominated communities. Experimental social scientists sometimes explicitly distinguish these two aspects of the decision faced by participants in CPR games—the “effort decision” regarding the best overall level of extraction to exert, and the “spatial decision” regarding how best to distribute this effort among multiple resources^13,14^—and seek to identify which aspect is more relevant to real-world CPR users’ self-regulation. Since both the CPR model at hand and typical PGG models rely on an assumption that one of these aspects of agents’ choice is fixed while the other is allowed to vary, neither model is able to directly address these issues on its own. We believe that our results, by highlighting the potential importance of allocation decisions in self-regulation, prompt further research into how the conclusions of each these models are altered if both “spatial” and “effort” decisions operate simultaneously. Alternatively, if real-world CPR agents are indeed found to exhibit some innate inclination or aversion toward reallocation, then our result prompts further investigations into how either of these bounds to agents’ rationality might itself have emerged. Whether these bounds arise from agents’ intrinsic cognitive limitations or from external factors which limit agents’ access to information about resource conditions, or might be “learned” or transmitted socially, a deeper understanding of these issues seems necessary to determine the applicability of these network evolutionary game models to real-world public goods or CPR dilemmas.

Aside from this parallel, the current CPR model differs from typical network PGG models in that it does not rely on any assumption that *imitation* drives agents’ strategy choices. In those models, cooperative strategies persist *only* because individuals—in lieu of direct knowledge of the actual payoff functions that map their actions onto their outcomes—*misattribute* the higher payoffs of their cooperator neighbors to the cooperative strategy itself, and so to imitate this strategy in hopes of achieving similar payoffs for themselves. Here, however, no such misunderstanding or miscommunication is required. In this CPR model, no single fixed extraction strategy is labelled as “cooperation”; rather, effective coordination among individuals depends on a population’s ability to adapt their strategies to collectively mitigate CPR degradation by chasing their own short-term interests based on accurate information about resource conditions. The results thus provide some insight into how agents might initially be incentivized to discover and practice more efficient patterns of extraction, rather than into how these patterns might then become established in the form of social norms and rules. Indeed, real-world CPR users are known to exchange information about resource conditions and intentions, and to coordinate their actions by negotiating and enforcing rules, via social networks^3^. We believe that the findings presented here might provide a more fundamental basis upon which to build these more elaborate networked CPR models, which incorporate additional social processes which interact with agents’ myopic self-interest to facilitate or hinder effective CPR self-regulation by reallocation.

Finally, we expect that it will be fruitful to explore potential links between the present work, which is rooted in a more abstract, evolutionary perspective, and the wider body of ongoing applied research that applies the tools of rational queueing theory^33,34^, dynamic pricing^35–37^, and social choice theory^38^ to address issues of resource allocation in networked systems^39–42^. Although the CPR literature may seem to suggest that regulation by a centralized decision-maker is likely doomed to failure, a study of the mechanisms underlying CPR self-regulation may help to inform research undertaken from an optimal control perspective, with the aim of identifying potential targeted interventions that respect and leverage a community’s innate capacity for bottom-up self-regulation rather than disrupt it. Introducing some form of dynamic pricing into these networked models^43,44^, for example, might represent one less-intrusive approach through which agents’ myopic self-interest could be redirected towards collectively beneficial outcomes while still being informed by changing resource conditions at the local level. Conversely, the specific game-theoretical formalism already adopted in application-oriented studies could also help to guide future extensions of more abstract models such as that of the present work. Studies of the collective dynamics of agents attempting to coordinate resource usage within these networks, while also perhaps paying for access to new resources in ways that alter the network structures themselves, might be informed by recent game-theoretical studies of hybrid public–private parking systems^45^. Models that allow agents to consider the longer-term, "non-myopic" consequences of decisions may also benefit from recent modelling work on transport services^46^.

## Supplementary information

Supplementary Information.
