# Supplementary material for: Myopic reallocation of extraction improves collective outcomes in networked common-pool resource games
Source: Sci Rep. 2021 Jan 13;11:886. doi: 10.1038/s41598-020-79514-5 (PMC7806614; doi:10.1038/s41598-020-79514-5)
Supplement: Supplementary file 1 — Supplementary Information. [file 41598_2020_79514_MOESM1_ESM.pdf]

Myopic reallocation of extraction improves collective outcomes in networked common-pool resource games: Supplemental Materials

Andrew Schauf[1,*] **and Poong Oh**[1]

[1]Nanyang Technological University, Wee Kim Wee School of Communication and Information, Singapore, 639798, Singapore
[*]andrew.schauf@ntu.edu.sg

SUMMARY

These Supplemental Materials provide additional details and results to support the article "Myopic reallocation of extraction improves collective outcomes in networked common-pool resource games". In Section S1, we introduce notations and detail the common-pool resource (CPR) reallocation game model and methods used. In Section S2, we discuss steady states of this evolutionary CPR reallocation game, proving that populations attain greater collective wealth in these steady states than in the initial conditions from which they evolve. Next, we derive estimates for the expected total extraction from CPR sources, agent extraction levels, and agent payoffs as functions of degree for Nash equilibrium and Pareto efficient states in Section S3. Section S4 then presents these estimates for a *degree-proportional capacity* scenario to supplement the corresponding results for a *uniform capacity* scenario presented in the article, demonstrating that the main findings reported in the article are robust with respect to this change in the dependence of CPR degradation characteristics upon degree. In Section S5, we detail the methods used to compute Nash equilibrium states, efficient states, and steady states of reallocation dynamics for specific networks. Finally, in Section S6, those methods are used to compute ensemble mean values of quantities for comparison to the heterogeneous mean-field estimates presented in the article, validating the estimates while also highlighting some of their limitations.

Contents

S1 Notations and model **2**
S1.1 Agent-source affiliation networks . . . 2
S1.2 Common-pool resource extraction game . . . 2
S1.3 Common-pool resource reallocation dynamics . . . 3
S2 Steady states of reallocation dynamics **3**
S2.1 Proof: Reallocation dynamics increase collective wealth . . . 3
S2.2 Steady states of reallocation dynamics are distinct from Nash equilibria . . . 4
S3 Estimating source extraction pressure and agent fitness by degree **4**
S3.1 Nash equilibrium states . . . 5
Cost-free extraction • Costly extraction • Degree dependence of payoff shifts under reallocation dynamics
S3.2 Pareto efficient states . . . 8
Cost-free extraction • Costly extraction
S4 Results: degree-proportional capacity scenario **9**
S5 Computing extraction states on networks **10**
S5.1 Computing steady states of reallocation dynamics . . . 10
S5.2 Computing Nash equilibrium states . . . 13
S5.3 Computing Pareto efficient states . . . 13
S6 Validation **14**
References **15**

S1 Notations and model

S1.1 Agent-source affiliation networks

We consider populations $\mathbf{A} = \{a_1, \ldots, a_M\}$ of M agent nodes and sets of N CPR source nodes $\mathbf{S} = \{s_1, \ldots, s_N\}$, where agents' access to sources are described by bipartite graphs $g = (\mathbf{A}, \mathbf{S}, \mathbf{L})$, in which the presence of an edge $(a, s) \in \mathbf{L}$ indicates that the agent a has access to source s. We denote the set of sources affiliated with an agent a as $\mathbf{S}_a = \{s \mid (a, s) \in \mathbf{L}\}$, and likewise for the population of agents affiliated with a source s: $\mathbf{A}_s = \{a \mid (a, s) \in \mathbf{L}\}$. We denote the network degree of an agent node a (its number of affiliated sources) as $m(a) = \|\mathbf{S}_a\|$, and the degree of a source node s (its number of affiliated agents) as $n(s) = \|\mathbf{A}_s\|$. We denote the maximum degrees of nodes of each type as $n_{\max} = \max_{s\in\mathbf{S}} n(s)$ and $m_{\max} = \max_{a\in\mathbf{S}} m(a)$. The network's distribution of agent degrees is denoted by $P_{\mathbf{A}}(m)$ and the distribution of source degrees by $P_{\mathbf{S}}(n)$. Conditional degree distributions are denoted as $P_{\mathbf{A}}(m \mid n)$ and $P_{\mathbf{S}}(n \mid m)$, respectively.

Networks were generated by first generating a prospective CPR source degree sequence from a given distribution: either a delta-function ("D") distribution, a normal distribution ("N"), or a power-law distribution ("PL"). The resulting degree sequence was accepted if its mean degree was equal to the desired target value $\langle n \rangle$, and otherwise the process was repeated. The set of N source nodes were then randomly linked to the M agent nodes in accord with the generated source degree sequence to yield a network with a random ("R") agent degree distribution. These networks with random agent degree distributions were then rewired to produce uniform ("U") agent degree distributions by randomly rewiring edges for agent nodes having current degree $m(a) > \langle m \rangle$ to those having $m(a) < \langle m \rangle$ until all agent nodes share uniform degree $m(a) \equiv \langle m \rangle$. An ensemble of networks with scale-free-type ("SF") agent degree distributions were also produced by applying a preferential attachment algorithm[1] to the original ensemble of networks with random agent degree distributions. The networks generated here all share the same total number of agent nodes $M = 50$, number of source nodes $N = 50$, and mean degrees $\langle m \rangle = \langle n \rangle = 5$, and are all restricted such that $m(a) \geq 2$ for all $a \in \mathbf{A}$, and $n(s) \geq 2$ for all $s \in \mathbf{S}$. Results are shown for ensembles of 10^3 agent-resource affiliation networks each of 9 types, each representing a combination of one of three types of source degree heterogeneity (Delta-function, Normal, or Power-Law) with one of three similar types of agent degree heterogeneity (Uniform, Random, or Scale-Free-type). From the resulting ensembles, we extract degree histograms which are averaged to yield representative degree distributions $P_{\mathbf{A}}(m)$ and $P_{\mathbf{S}}(n)$ for each network type (Figure 1); these are used to compute heterogeneous mean-field estimates of various quantities of interest as discussed below.

S1.2 Common-pool resource extraction game

Following and generalizing upon the approach of İlkılıç[2], we consider the extraction game in which each agent a exerts an effort of magnitude $q(a, s)$ towards extraction from an affiliated source s; an agent's strategy thus comprises its choices of $q(a, s)$ for all of its affiliated sources $s \in \mathbf{S}_a$. We denote the total extraction effort exerted by an agent as $\overleftarrow{q}(a) = \sum_{s\in\mathbf{S}_a} q(a, s)$, which we will refer to as the agent's *extraction intensity*, and the total extraction effort exerted upon a source by $\overrightarrow{q}(s) = \sum_{a\in\mathbf{A}_s} q(a, s)$, which we will refer to as the *extraction pressure* on source s. We define the *quality*, $b(s)$, of a source s as the magnitude of the benefit it offers to a user in return for each unit of pressure exerted upon it. The total fitness of an agent a is thus given by the payoff function

$$f(a) = \left[\sum_{s\in\mathbf{S}_a} q(a, s) \cdot b(s)\right] - \frac{\gamma(a)}{2} \overleftarrow{q}(a)^2, \tag{S1}$$

where the constant $\gamma(a) > 0$ in the quadratic cost term quantifies the relative strength of diminishing marginal utility for the agent a. We model the rivalrous (or *subtractable*) nature of CPR sources by assuming that each source s offers some basic benefit per unit extraction, $\alpha(s)$, which is reduced by some amount $\beta(s)$ for each unit of extraction pressure the source receives, so that

$$b(s) = \alpha(s) - \beta(s) \overrightarrow{q}(s). \tag{S2}$$

In these Supplemental Materials, unlike the main article, we consider a more general case which allows for variability among the values of the parameter $\gamma(a)$ for each agent $a \in \mathbf{A}$ and the values of $\alpha(s)$ for each source $s \in \mathbf{S}$, in addition to the variability of $\beta(s)$ considered in the article itself. In the computations presented in the article and the materials below (Section S4 and Section S6), we present results from both a *uniform capacity* scenario where $\beta(s) = \beta_0$ for some constant $\beta_0 > 0$ across all sources $s \in \mathbf{S}$, and also for a *degree-proportional capacity* scenario where $\beta(s) = \beta_0 \langle n \rangle / n(s)$, so that sources degrade in quality in proportion to their total extraction pressure *per user*. When considering parameters as functions of node degree rather than of individual nodes, as in the degree-binned estimates of Section S3, we will denote the degree dependence using subscripts: α_n, β_n, and γ_m.

S1.3 Common-pool resource reallocation dynamics

Here we consider the scenario wherein agents are allowed to reallocate their extraction efforts among the multiple sources to which they have access, with the rate of reallocation from one source to another being proportional to the difference in quality between them, such that

$$\frac{\mathrm{d}}{\mathrm{d}t} q(a,s) = k \sum_{s' \in \mathbf{S}_a} \left[b(s) - b(s') \right] , \tag{S3}$$

where $k > 0$ is some constant. These reallocation dynamics conserve each agent's total extraction intensity $\overleftarrow{q}(a)$ in time since $\frac{\mathrm{d}}{\mathrm{d}t}\overleftarrow{q}(a) = \frac{\mathrm{d}}{\mathrm{d}t}\left(\sum_{s\in\mathbf{S}_a} q(a,s)\right) = k\sum_{s\in\mathbf{S}_a}\sum_{s'\in\mathbf{S}_a}[b(s)-b(s')] = 0$.

S2 Steady states of reallocation dynamics

S2.1 Proof: Reallocation dynamics increase collective wealth

In this section, we confirm that reallocation dynamics described by Equation S3 will increase a population's total collective wealth in the CPR extraction game described above. Steady states of the reallocation dynamics described by Equation S3 are those in which all sources share a common final quality value, which we denote as b_f, and so have $\frac{\mathrm{d}}{\mathrm{d}t} q(a,s) = 0$ for all extraction levels $q(a,s)$. Using Equation S2, the population's total extraction Q at steady state can be expressed in terms of b_f as

$$Q = \sum_{s\in\mathbf{S}} \overrightarrow{q}(s) = \sum_{s\in\mathbf{S}} \frac{1}{\beta(s)} \left[\alpha(s) - b_f \right] = N \left[\langle \alpha\beta^{-1} \rangle - b_f \langle \beta^{-1} \rangle \right] , \tag{S4}$$

where brackets indicate an expected value of a function of source node s over the set of all sources: $\langle x \rangle = \left(\sum_{s\in\mathbf{S}} x(s)\right)/N$. Rearranging Equation S4, it follows that the steady-state quality b_f of each source is given in terms of total extraction Q as

$$b_f = \frac{\langle \alpha\beta^{-1} \rangle - QN^{-1}}{\langle \beta^{-1} \rangle} . \tag{S5}$$

The steady-state extraction value Q also represents total extraction in the initial condition under consideration, since the dynamics of Equation S3 conserve this value over time. Similarly, the quadratic cost term $\gamma\overleftarrow{q}(a)^2/2$ in Equation S1 is unaffected by reallocation dynamics, which conserve the magnitudes of $\overleftarrow{q}(a)$ for all $a \in \mathbf{A}$. At steady state, the population's total benefits received (ignoring these unchanging cost terms) is $F_f = \sum_{s\in\mathbf{S}} \left[\overrightarrow{q}(s) \cdot b_f\right] = Qb_f$. Reallocation dynamics thus lead to an improved overall fitness if $F_0 < F_f$, that is, if $F_0/Q < b_f$, where $F_0 = \sum_{s\in\mathbf{S}} \overrightarrow{q}(s) \cdot b(s)$ is the total collective payoff of the initial condition. The ratio F_0/Q is given by

$$\frac{F_0}{Q} = \frac{\sum_{s\in\mathbf{S}} \overrightarrow{q}(s) \cdot [\alpha(s) - \beta(s)\overrightarrow{q}(s)]}{\sum_{s\in\mathbf{S}} \overrightarrow{q}(s)} = \frac{\langle \overrightarrow{q} \cdot [\alpha - \beta\overrightarrow{q}] \rangle}{\langle \overrightarrow{q} \rangle} . \tag{S6}$$

Using Equations S5 and S6, the condition $F_0/Q < b_f$ can be expressed as

$$\frac{\langle \alpha\overrightarrow{q} \rangle - \langle \beta\overrightarrow{q}^2 \rangle}{\langle \overrightarrow{q} \rangle} < \frac{\langle \alpha\beta^{-1} \rangle - \langle \overrightarrow{q} \rangle}{\langle \beta^{-1} \rangle} . \tag{S7}$$

Assuming that $\alpha(s)$ is uncorrelated with $\beta(s)$ and $\overrightarrow{q}(s)$ (as in all scenarios considered in this article, where $\alpha(s) \equiv \alpha_0$ for all $s \in \mathbf{S}$), the condition that $F_0 < F_f$ further reduces to

$$\langle \overrightarrow{q} \rangle^2 < \langle \beta\overrightarrow{q}^2 \rangle \langle \beta^{-1} \rangle . \tag{S8}$$

These reallocation dynamics will thus improve collective wealth if and only if this inequality holds.

Indeed, the validity of Equation S8 is guaranteed by the Cauchy-Schwarz inequality[3], which states, for random variables X and Y, that $\langle XY \rangle^2 \le \langle X^2 \rangle \langle Y^2 \rangle$. With the identifications $X = \sqrt{\beta(s)}\overrightarrow{q}(s)$ and $Y = \sqrt{\beta(s)^{-1}}$, then, we have

$$\langle \overrightarrow{q} \rangle^2 = \left\langle \left[\sqrt{\beta}\overrightarrow{q}\right] \left[\sqrt{\beta^{-1}}\right] \right\rangle^2 \le \langle \beta\overrightarrow{q}^2 \rangle \langle \beta^{-1} \rangle , \tag{S9}$$

and thus $F_0 \le F_f$ by the Cauchy-Schwarz inequality. Furthermore, equality in Equation S9 occurs if and only if $X/Y = K$ for some constant K[3], that is, when $\beta(s)\overrightarrow{q}(s) \equiv K$ for all sources $s \in \mathbf{S}$. This indeed describes the steady states of reallocation dynamics, in which all sources share a uniform quality value: $b_f = \alpha_0 - \beta(s)\overrightarrow{q}(s) = \alpha_0 - K$. This proves that the reallocation dynamics of Equation S3 will improve collective wealth in the CPR extraction game for all initial extraction states that are not already characterized by uniform source quality.

S2.2 Steady states of reallocation dynamics are distinct from Nash equilibria

We now clarify the relationship of these steady states to the notion of Nash equilbrium. Nash equilibria of the extraction game represent best-response extraction levels that maximize each individual's payoffs provided that all others are attempting to do the same (see Section S5.2 below). In Nash equilibrium states, each agent sets its extraction levels at each of its affiliated sources such that any further extraction would increase costs and reduce source quality to an extent that outweighs the additional benefits extracted thereby. If all other extraction levels are held constant, then, any alteration that an agent might make to its extraction levels would reduce its payoffs. İlkılıç showed that any given network has a *unique* Nash equilibrium within the network CPR extraction game generalized in Section S1.2 above[2].

In the current work, we consider the dynamics that occur when agents are restricted to perform only *reallocation* updates that shift extraction effort among their affiliated sources while conserving their overall magnitudes of extraction effort. Steady states of reallocation dynamics (Equation S3) are those in which all sources having some affiliated agents in common share an identical quality value, and so agents perceive no incentive to reallocate. The unique Nash equibrium states of the CPR extraction game typically show unequal quality among sources (see Section S5.2 below), and so are *not* steady states of these reallocation dynamics (in contrast with Pareto efficient states, which in the games considered here *are* also steady states; see Section S3.2 below). We now address the question of whether these steady states coincide with a notion of Nash equilibrium that incorporates the additional constraints introduced in a *CPR reallocation game*: do there exist any *reallocations* which an agent could perform that would increase its own payoff given that all other extraction levels were held constant? Below, we confirm that these potential reallocations typically *do* exist at steady states.

Consider a steady state of reallocation dynamics wherein all sources share a common quality value, b_f. A *reallocation* is an update by which an agent reduces extraction at one of its affiliated sources s_1 by some amount ε, while also increasing its extraction at another affiliated source s_2 by an equal amount. Based on Equation S1, the agent's payoff in the steady state is

$$f_{\mathrm{SS}}(a) = q(a,s_1)b_f + q(a,s_2)b_f + \left[\sum_{s'\in\mathbf{S}_a\setminus\{s_1,s_2\}} q(a,s')\cdot b_f\right] - \frac{\gamma}{2}\overleftarrow{q}(a)^2. \tag{S10}$$

After reallocation, since source quality varies as in Equation S2, the agent's payoff becomes

$$f_{\mathrm{R}}(a) = (q(a,s_1)-\varepsilon)\left[b_f+\beta\varepsilon\right] + (q(a,s_2)+\varepsilon)\left[b_f-\beta\varepsilon\right] + \left[\sum_{s'\in\mathbf{S}_a\setminus\{s_1,s_2\}} q(a,s')\cdot b_f\right] - \frac{\gamma}{2}\overleftarrow{q}(a)^2. \tag{S11}$$

Given that all other extraction levels remain constant, the reallocation shifts the agent's payoff by

$$f_{\mathrm{R}}(a) - f_{\mathrm{SS}}(a) = \beta\varepsilon\left[q(a,s_1)-q(a,s_2)\right] - 2\beta\varepsilon^2. \tag{S12}$$

In general, $f_{\mathrm{R}}(a) - f_{\mathrm{SS}}(a) \neq 0$, and in the limit $\varepsilon \to 0$ reallocations improve fitness when

$$\lim_{\varepsilon\to 0}\left[\frac{f_{\mathrm{R}}(a)-f_{\mathrm{SS}}(a)}{\varepsilon}\right] = \beta\left[q(a,s_1)-q(a,s_2)\right] > 0. \tag{S13}$$

This demonstrates that reallocations that shift the agent's effort away from sources where the agent currently exerts greater extraction effort towards sources where it exerts less effort – redistributing an agent's own *effort* more evenly among its affiliated sources rather than equalizing the *quality* of all affiliated sources – could improve an agent's fitness if performed unilaterally while all other agents held their extraction levels constant. However, for the boundedly rational agents engaging in this reallocation game, the assumption that next-round payoffs are increased only by shifting effort from lower- to higher-quality sources (that is, the implicit assumption that source quality values will remain unchanged in the subsequent round, unaffected even by the agent's own reallocations) prevent them from enacting such an update. So, in general, the steady states of the reallocation dynamics (Equation S3) are *not* Nash equilbria of the CPR extraction game, nor do they coincide with the aforementioned notion of Nash equilibrium within the more specific context of the CPR reallocation game.

S3 Estimating source extraction pressure and agent fitness by degree

We will now derive estimates for the expected degree dependence of CPR source extraction and agent fitness in Nash equilibrium and efficient states given a network's degree distributions. The heterogeneous mean-field-type approach detailed below is used to compute the results shown in Figure 2, Figure 3, and Figure 4 in the article, as well as Figure S1, Figure S2, and Figure S3 in the sections below.

S3.1 Nash equilibrium states

Nash equilibrium describes the best-response choices of strategies $q(a,s)$ when all agents simultaneously seek to maximize their own payoffs $f(a)$. The equilibrium condition[2] $\frac{\partial f(a)}{\partial q(a,s)} = 0$ (applied to Equation S1 with source quality specified by Equation S2) restricts each nonzero $q(a,s)$ to satisfy

$$q(a,s) = \frac{\alpha(s)}{\beta(s)} - \overrightarrow{q}(s) - \frac{\gamma(a)}{\beta(s)}\overleftarrow{q}(a). \tag{S14}$$

Summing this condition over all agents $\mathbf{A}_s$ affiliated with a particular source s yields

$$\overrightarrow{q}(s) = -n(s)\overrightarrow{q}(s) + n(s)\left(\frac{\alpha(s)}{\beta(s)}\right) - \frac{1}{\beta(s)}\left(\sum_{a\in\mathbf{A}_s}\gamma(a)\overleftarrow{q}(a)\right), \tag{S15}$$

which we rearrange to express the source's total extraction pressure at equilibrium as

$$\overrightarrow{q}(s) = \frac{1}{\beta(s)}\left[\frac{n(s)}{n(s)+1}\right]\left[\alpha(s) - \frac{1}{n(s)}\left(\sum_{a\in\mathbf{A}_s}\gamma(a)\overleftarrow{q}(a)\right)\right]. \tag{S16}$$

We now use Equation S16 to estimate the degree dependence of agents' expected extraction levels and payoffs for the cases of cost-free extraction ($\gamma(a)\equiv 0$), which we compute directly using degree distributions, and costly extraction with diminishing marginal utility ($\gamma(a) > 0$), which we compute numerically as the solution of a linear system as detailed below.

S3.1.1 Cost-free extraction

In the case of cost-free extraction ($\gamma(a)\equiv 0$), Equation S16 reduces to

$$\overrightarrow{q}(s) = \frac{\alpha(s)}{\beta(s)}\left[\frac{n(s)}{n(s)+1}\right], \tag{S17}$$

so that Nash equilibrium source quality is given by

$$b(s) = \alpha(s)\left[\frac{1}{n(s)+1}\right] \tag{S18}$$

Focusing first on the population's total collective extraction and payoffs, by Equation S17 the population's total collective extraction is

$$Q = N\sum_{n=1}^{n_{\max}} P_{\mathbf{S}}(n)\cdot\overrightarrow{q}(s) = N\left\langle\frac{\alpha_n}{\beta_n}\left[\frac{n}{n+1}\right]\right\rangle, \tag{S19}$$

where brackets around variables with subscript n indicate expected values $\langle x_n\rangle = \sum_{n=1}^{n_{\max}} P_{\mathbf{S}}(n)\cdot x_n$. Using Equations S17 and S18, this extraction leads the population to derive total collective wealth

$$F = N\sum_{n=1}^{n_{\max}} P_{\mathbf{S}}(n)\cdot[\overrightarrow{q}(s)\cdot b(s)] = N\left\langle\frac{\alpha_n^2}{\beta_n}\left[\frac{n}{(n+1)^2}\right]\right\rangle. \tag{S20}$$

Total equilibrium extraction and wealth will thus both be larger for networks with greater source degree heterogeneity in the uniform capacity scenario ($\beta_n\equiv\beta_0$), and smaller for networks with greater source degree heterogeneity in the degree-proportional capacity scenario ($\beta_n = \beta_0\langle n\rangle/n$).

Now focusing on the extraction and payoffs of individual agents as functions of their network degrees, we now estimate the total extraction effort $\overleftarrow{q}(s)$ of an agent a with degree m at Nash equilibrium. Using Equation S17, which in light of Equation S14 gives $q(a,s) = \overrightarrow{q}(s)/n(s)$, we estimate

$$\langle\overleftarrow{q}\rangle_m = m\sum_{n=1}^{n_{\max}} P_{\mathbf{S}}(n\mid m)\cdot q(a,s) = m\left\langle\frac{\alpha_n}{\beta_n}\left[\frac{1}{n+1}\right]\right\rangle_m \tag{S21}$$

$$= m\frac{1}{\langle n\rangle}\left\langle\frac{\alpha_n}{\beta_n}\left[\frac{n}{n+1}\right]\right\rangle = \frac{m}{N\langle n\rangle}Q, \tag{S22}$$

where the subscripted brackets indicate an expected value over the conditional degree distribution, $\langle x_n \rangle_m = \sum_{n=1}^{n_{\max}} P_{\mathbf{S}}(n \mid m) \cdot x_n$, and in the last two steps we have invoked an additional assumption that the network is formed without any degree-based preferential linking tendencies so that we can accurately approximate $P_{\mathbf{S}}(n \mid m) = P_{\mathbf{S}}(n)\, n / \langle n \rangle$. In the absence of degree-degree correlations and diminishing marginal utility, then, an individual agent's fraction of the population's total extraction at equilibrium is simply equal to the fraction of the network's total $N\langle n \rangle$ edges that it possesses. Similarly for a degree-m agent's expected payoffs, now using Equations S17 and S18 we find

$$\langle f \rangle_m = m \sum_{n=1}^{n_{\max}} P_{\mathbf{S}}(n \mid m) \cdot [q(a,s) \cdot b(s)] = m \sum_{n=1}^{n_{\max}} P_{\mathbf{S}}(n \mid m) \cdot \left(\frac{\alpha_n^2}{\beta_n} \left[\frac{1}{(n+1)^2} \right] \right) = m \left\langle \frac{\alpha_n^2}{\beta_n} \left[\frac{1}{(n+1)^2} \right] \right\rangle_m \tag{S23}$$

$$= m \frac{1}{\langle n \rangle} \left\langle \frac{\alpha_n^2}{\beta_n} \left[\frac{n}{(n+1)^2} \right] \right\rangle = \frac{m}{N\langle n \rangle} F, \tag{S24}$$

where again the last two steps hold in the absence of degree-degree correlations.

S3.1.2 Costly extraction

In order to estimate expected extraction pressure $\langle \overrightarrow{q} \rangle_n$ and expected agent extraction $\langle \overleftarrow{q} \rangle_m$ and payoff $\langle f \rangle_m$ using degree distributions in the case of costly extraction ($\gamma_m > 0$), we construct and solve a linear system. First, we sum the equilibrium condition of S14 over all sources $s \in \mathbf{S}_a$ affiliated with an agent a, which yields

$$\overleftarrow{q}(a) = -\gamma(a) \left(\sum_{s \in \mathbf{S}_a} \frac{1}{\beta(s)} \right) \overleftarrow{q}(a) + \left(\sum_{s \in \mathbf{S}_a} \frac{\alpha(s)}{\beta(s)} \right) - \left(\sum_{s \in \mathbf{S}_a} \overrightarrow{q}(s) \right). \tag{S25}$$

In degree-statistical form (in accord with a heterogeneous mean-field perspective), for a degree-m agent this becomes

$$\langle \overleftarrow{q} \rangle_m = -\gamma_m m \langle \beta_n^{-1} \rangle_m \langle \overleftarrow{q} \rangle_m + m \langle \alpha_n \beta_n^{-1} \rangle_m - m \left(\sum_{n=1}^{n_{\max}} P_{\mathbf{S}}(n \mid m) \cdot \langle \overrightarrow{q} \rangle_n \right), \tag{S26}$$

which we rearrange to estimate the total extraction of a degree-m agent in terms of expected extraction pressures $\langle \overrightarrow{q} \rangle_n$ as

$$\langle \overleftarrow{q} \rangle_m = \left(\frac{m}{m\gamma_m \langle \beta^{-1} \rangle_m + 1} \right) \left[\langle \alpha \beta^{-1} \rangle_m - \left(\sum_{n=1}^{n_{\max}} P_{\mathbf{S}}(n \mid m) \cdot \langle \overrightarrow{q} \rangle_n \right) \right]. \tag{S27}$$

Expressing the source extraction pressure of Equation S16 in a degree-statistical form, we write

$$\langle \overrightarrow{q} \rangle_n = \frac{1}{\beta_n} \left[\frac{n}{n+1} \right] \left[\alpha_n - \left(\sum_{m=1}^{m_{\max}} P_{\mathbf{A}}(m \mid n) \cdot [\gamma_m \langle \overleftarrow{q} \rangle_m] \right) \right]. \tag{S28}$$

By substitution using Equation S27 to express the values $\langle \overleftarrow{q} \rangle_m$ in terms of extraction pressures $\langle \overrightarrow{q} \rangle_{n'}$, Equation S28 becomes

$$\langle \overrightarrow{q} \rangle_n = \frac{1}{\beta_n} \left[\frac{n}{n+1} \right] \left[\alpha_n - \sum_{m=1}^{m_{\max}} P_{\mathbf{A}}(m \mid n) \cdot \left(\frac{m\gamma_m}{[m\gamma_m \langle \beta^{-1} \rangle_m + 1]} \left[\langle \alpha \beta^{-1} \rangle_m - \sum_{n'=1}^{n_{\max}} P_{\mathbf{S}}(n' \mid m) \cdot \langle \overrightarrow{q} \rangle_{n'} \right] \right) \right]. \tag{S29}$$

The system of conditions defined by Equation S29, with one for each source degree $n \in \{1, 2, \ldots, n_{\max}\}$ represented in the network, constitute a linear system

$$\mathbf{C}\tilde{\mathbf{Q}} = \tilde{\mathbf{y}}, \tag{S30}$$

where the vector $\tilde{\mathbf{Q}}$ has entries $Q_n = \langle \overrightarrow{q} \rangle_n$, the matrix $\mathbf{C}$ has entries

$$C_{n,n'} = -\frac{1}{\beta_n} \left[\frac{n}{n+1} \right] \sum_{m=1}^{m_{\max}} P_{\mathbf{A}}(m \mid n) \cdot \left(\left[\frac{m\gamma_m}{m\gamma_m \langle \beta^{-1} \rangle_m + 1} \right] P_{\mathbf{S}}(n' \mid m) \right), \tag{S31}$$

and the vector $\tilde{\mathbf{y}}$ has entries

$$y_n = \frac{1}{\beta_n} \left[\frac{n}{n+1} \right] \left[\alpha_n - \left(\sum_{m=1}^{m_{\max}} P_{\mathbf{A}}(m \mid n) \cdot \left[\frac{m\gamma_m \langle \alpha \beta^{-1} \rangle_m}{m\gamma_m \langle \alpha \beta^{-1} \rangle_m + 1} \right] \right) \right]. \tag{S32}$$

Given degree distributions $P_{\mathbf{A}}(m \mid n)$ and $P_{\mathbf{S}}(m \mid n)$ representing a given network type, we compute $\tilde{\mathbf{Q}} = \mathbf{C}^{-1}\tilde{\mathbf{y}}$ numerically (here, we use Python 3.7.3 with SciPy 1.2.1[4]) to yield values for $\langle \overrightarrow{q} \rangle_n$. Along with the values of $\langle \overleftarrow{q} \rangle_m$ then computed by inserting these values of $\langle \overrightarrow{q} \rangle_n$ into Equation S27, the expected extraction by a degree-m agent from a degree-n source, $\langle q \rangle_{m,n}$, can then be estimated based on the Nash equilibrium condition of Equation S14 as

$$\langle q \rangle_{m,n} = \frac{\alpha_n}{\beta_n} - \langle \overrightarrow{q} \rangle_n - \frac{\gamma_m}{\beta_n} \langle \overleftarrow{q} \rangle_m. \tag{S33}$$

Using these values of $\langle q \rangle_{m,n}$, along with the values of $\langle \overrightarrow{q} \rangle_n$ computed as solutions to the system of Equation S30, and the values of $\langle \overleftarrow{q} \rangle_m$ from Equation S27, the expected Nash equilibrium payoff $\langle f \rangle_m$ for a degree-m agent based on the payoff function of Equation S1 is estimated as

$$\langle f \rangle_m = m \sum_{n=1}^{n_{\max}} P_{\mathbf{S}}(n \mid m) \cdot \left[\langle q \rangle_{m,n} \left(\alpha_n - \beta_n \langle \overrightarrow{q} \rangle_n \right) \right] - \frac{\gamma_m}{2} \langle \overleftarrow{q} \rangle_m^2. \tag{S34}$$

The population's total extraction Q can then be computed using the solutions of Equation S30 as

$$Q = N \sum_{n=1}^{n_{\max}} P_{\mathbf{S}}(n) \cdot \langle \overrightarrow{q} \rangle_n, \tag{S35}$$

and total collective wealth F can be computed using Equation S34 as

$$F = M \sum_{m=1}^{m_{\max}} P_{\mathbf{A}}(m) \cdot \langle f \rangle_m, \tag{S36}$$

These estimates of extraction pressure as a function of source degree, $\langle \overrightarrow{q} \rangle_n$ are compared to the corresponding estimates from steady states to compute the estimated changes in extraction pressure shown in Figure 3a, Figure 4a, Figure S2a, and Figure S3a. Expected payoff values $\langle f \rangle_m$ (Equation S28) are similarly used to compute the estimated changes in payoffs shown in Figure 3b, Figure 4b, Figure S2b, and Figure S3b. These fitness estimates are also used to compute Gini index values (labelled as G_{Eq}) shown in Figure 2c-d, and Figure S1c-d using

$$G = \frac{1}{2\mu} \sum_{m_1=1}^{m_{\max}} \sum_{m_2=1}^{m_{\max}} P_{\mathbf{A}}(m_1) P_{\mathbf{A}}(m_2) \cdot \left| \langle f \rangle_{m_1} - \langle f \rangle_{m_2} \right|, \tag{S37}$$

where $\mu = \sum_{m=1}^{m_{\max}} P_{\mathbf{A}}(m) \cdot \langle f \rangle_m$. The values of total wealth computed by Equation S36 (labelled as F_{Eq}) are compared to those from efficient states computing using degree distributions for each network type to determine the efficiency values shown in Figure 2a-b and Figure S1a-d. Methods used to compute Nash equilibrium extraction states for a specific network realization are detailed below in Section S5.2.

S3.1.3 Degree dependence of payoff shifts under reallocation dynamics

Now we discuss the dependence of the expected payoffs shifts $\Delta\langle f \rangle_m$ upon agent degree m that occur under reallocation dynamics from Nash equilibrium (as displayed in Figure 3b, Figure 4b, Figure S2b and Figure S3b). If we denote the expected quality of a degree-n source at Nash equilibrium as $\langle b \rangle_n = \alpha_n - \beta_n \langle \overrightarrow{q} \rangle_n$, and use Equation S33 to estimate the expected extraction effort by a degree-m agent from a degree-n source at Nash equilibrium, $\langle q \rangle_{m,n}$, then the expected shift in payoffs experienced by a degree-m agent when all sources shift in quality towards a common steady-state value b_f is

$$\Delta\langle f \rangle_m = m \sum_{n=1}^{n_{\max}} P_{\mathbf{S}}(n \mid m) \cdot \left[\langle q \rangle_{m,n} \left(b_f - \langle b \rangle_n \right) \right] = m \sum_{n=1}^{n_{\max}} P_{\mathbf{S}}(n \mid m) \cdot \left[\frac{\langle b \rangle_n}{\beta_n} - \frac{\gamma_m}{\beta_n} \langle \overleftarrow{q} \rangle_m \right] \left(b_f - \langle b \rangle_n \right) \tag{S38}$$

$$= m \left[\left(\left\langle \frac{\langle b \rangle_n}{\beta_n} \right\rangle_m b_f - \left\langle \frac{\langle b \rangle_n^2}{\beta_n} \right\rangle_m \right) - \gamma_m \langle \overleftarrow{q} \rangle_m \left(\left\langle \frac{1}{\beta_n} \right\rangle_m b_f - \left\langle \frac{\langle b \rangle_n}{\beta_n} \right\rangle_m \right) \right] \tag{S39}$$

$$= m \left(\frac{1}{\langle n \rangle} \left[\left\langle \frac{n \langle b \rangle_n}{\beta_n} \right\rangle b_f - \left\langle \frac{n \langle b \rangle_n^2}{\beta_n} \right\rangle \right] \right) - \gamma_m m \langle \overleftarrow{q} \rangle_m \left(\frac{1}{\langle n \rangle} \left[\left\langle \frac{n}{\beta_n} \right\rangle - \left\langle \frac{n \langle b \rangle_n}{\beta_n} \right\rangle \right] \right) \tag{S40}$$

where the last step holds in the absence of degree-degree correlations. In the case of cost-free extraction ($\gamma_m \equiv 0$), the second term vanishes, and so in the absence of degree-degree correlations we expect $\Delta\langle f \rangle_m \propto m$, and the expected increase in payoff per source increases with the heterogeneity of the source degree distribution. For costly extraction ($\gamma_m > 0$), the magnitude of the second term grows with degree m as $m\langle \overleftarrow{q} \rangle_m$. Since $\langle \overleftarrow{q} \rangle_m$ itself increases with m, agents with higher degree – who are

incentivized by diminishing marginal utility to reduce their total amount of extraction per source $\langle \overleftarrow{q} \rangle_m / m$ at equilibrium – then as reallocation dynamics bring all sources to provide a uniform benefit per unit extraction, b_f, these higher-degree agents tend to find themselves extracting fewer of these redistributed benefits per source than do lower-degree agents. Lower-degree agents will then claim a larger relative fraction of the network's total wealth than they had at Nash equilibrium. This argument should hold more generally for initial conditions wherein agents' extraction intensity values tend to increase as concave functions of their degrees.

Because of this, reallocation dynamics from Nash equilibrium can increase the equality of the population's wealth distribution when payoff functions include diminishing marginal utility, but tend to preserve existing wealth inequality – at least insofar as it is based on degree – in the case of cost-free extraction. We note that wealth inequality may also be similarly preserved under certain other forms of costly extraction, for example when costs increase linearly with total extraction and so do not model diminishing marginal utility. For example, if the quadratic cost term in the payoff function of Equation S1 is replaced with a linear term, $\gamma(a)\overleftarrow{q}(a)^2/2 \to \gamma(a)\overleftarrow{q}(a)$, then the resulting Nash equilibrium condition gives an expression for extraction pressure which resembles that of the cost-free case in Equation S17, but with the substitution $\alpha(s) \to \alpha(s) - \langle \gamma(a) \rangle$, where $\langle \gamma(a) \rangle = \left(\sum_{a \in \mathbf{A}_s} \gamma(a) \right) / n(s)$. It follows that the Nash equilibrium expected agent extraction intensity $\langle \overleftarrow{q} \rangle_m$ corresponding to Equation S22 and expected fitness $\langle f \rangle_m$ corresponding to Equation S24 will both have a linear dependence on agent degree m, just as in the cost-free case discussed above. Steady states that evolve from Nash equilibrium initial conditions under reallocation dynamics will then similarly have $\langle f \rangle_m = \langle \overleftarrow{q} \rangle_m \cdot b_f \propto m$, so that reallocation dynamics will not affect the distribution of wealth among the population's different degree classes, just as in the cost-free case.

S3.2 Pareto efficient states

We now seek to estimate CPR source extraction and agent fitness given a network's degree distributions within Pareto efficient states, that is, states which maximize the population's total wealth $F = \sum_{a \in \mathbf{A}} f(a)$. In these states, each nonzero extraction value $q(a,s)$ satisfies $\frac{\partial F}{\partial q(a,s)} = 0$, and so satisfies a condition[2]

$$\gamma(a)\overleftarrow{q}(a) = \alpha(s) - 2\beta(s)\overrightarrow{q}(s). \tag{S41}$$

Shifting to a heterogeneous mean-field perspective, and assuming parameters are functions of node degree ($\alpha(s) = \alpha_{n(s)}$, $\beta(s) = \beta_{n(s)}$ and $\gamma(a) = \gamma_{m(a)}$), this becomes

$$\gamma_m \langle \overleftarrow{q} \rangle_m = \alpha_n - 2\beta_n \langle \overrightarrow{q} \rangle_n. \tag{S42}$$

We will now consider the cases of cost-free and costly extraction in turn.

S3.2.1 Cost-free extraction

When $\gamma_m \equiv 0$, then Equation S42 fixes

$$\langle \overrightarrow{q} \rangle_n = \frac{\alpha_n}{2\beta_n}, \tag{S43}$$

so that sources have quality $\langle b \rangle_n = \alpha_n/2$. By Equation S43, the population's total collective extraction in efficient states is then

$$Q = N \sum_{n=1}^{n_{\max}} P_{\mathbf{S}}(n) \cdot \langle \overrightarrow{q} \rangle_n = \frac{N}{2} \left\langle \frac{\alpha_n}{\beta_n} \right\rangle. \tag{S44}$$

Since the efficient-state extraction pressure of Equation S43 corresponds to source quality $\langle b \rangle_n = \alpha_n/2$, the maximum possible collective wealth attainable by the population is then

$$F = N \sum_{n=1}^{n_{\max}} P_{\mathbf{S}}(n) \cdot [\langle \overrightarrow{q} \rangle_n \langle b \rangle_n] = \frac{N}{4} \left\langle \frac{\alpha_n^2}{\beta_n} \right\rangle. \tag{S45}$$

In both the uniform capacity scenario ($\alpha_n \equiv \alpha_0$, $\beta_n \equiv \beta_0$) and the degree-proportional capacity scenario ($\alpha_n \equiv \alpha_0$, $\beta_n = \beta_0 \langle n \rangle / n$), we have $\langle \alpha_n \beta_n^{-1} \rangle = \alpha_0 \beta_0^{-1}$ and $\langle \alpha_n^2 \beta_n^{-1} \rangle = \alpha_0^2 \beta_0^{-1}$, and so the values of total extraction and wealth in efficient states do *not* depend on the network's degree distributions. While Equation S42 itself does not restrict agent extraction levels $\langle \overleftarrow{q} \rangle_m$, the expression for $\langle \overleftarrow{q} \rangle_m$ in the case of costly extraction (see Equation S52 below) taken in the cost-free limit $\gamma_m \to 0$ suggests that

$$\langle \overleftarrow{q} \rangle_m = \frac{N}{2M} \left\langle \frac{\alpha_n}{\beta_n} \right\rangle = \frac{Q}{M}, \tag{S46}$$

such that all agents exert the same magnitude of extraction effort $\langle \overleftarrow{q} \rangle_m$ regardless of their degrees. Agent payoffs also have an egalitarian distribution among all agents regardless of their degree in efficient states, as

$$\langle f \rangle_m = Q \langle b \rangle_n = \frac{N}{4M} \left\langle \frac{\alpha_n^2}{\beta_n} \right\rangle = \frac{F}{M}. \tag{S47}$$

S3.2.2 Costly extraction

When $\gamma_m > 0$, the lack of dependence of the left-hand side of Equation S42 on source degree n, and the lack of dependence of the right-hand side on agent degree m, implies that both sides must be equal to some constant K regardless of degrees m or n:

$$K \equiv \gamma_m \langle \overleftarrow{q} \rangle_m = \alpha_n - 2\beta_n \langle \overrightarrow{q} \rangle_n. \tag{S48}$$

Consistency between the sum of all extraction effort exerted by agents and the total extraction pressure applied to sources then requires

$$M \sum_{m=1}^{m_{\max}} P_{\mathbf{A}}(m) \cdot \langle \overleftarrow{q} \rangle_m = N \sum_{n=1}^{n_{\max}} P_{\mathbf{S}}(n) \cdot \langle \overrightarrow{q} \rangle_n, \tag{S49}$$

which if $\langle \overleftarrow{q} \rangle_m$ and $\langle \overrightarrow{q} \rangle_n$ are expressed in terms of K gives

$$MK \sum_{m=1}^{m_{\max}} P_{\mathbf{A}}(m) \cdot \left[\frac{1}{\gamma_m} \right] = N \sum_{n=1}^{n_{\max}} P_{\mathbf{S}}(n) \cdot \left[\frac{\alpha_n - K}{2\beta_n} \right]. \tag{S50}$$

Rearranging this, we find

$$K = \frac{\langle \alpha_n \beta_n^{-1} \rangle}{\langle \beta_n^{-1} \rangle + 2MN^{-1} \langle \gamma_m^{-1} \rangle} \tag{S51}$$

where $\langle \gamma_m^{-1} \rangle = \sum_{m=1}^{m_{\max}} P_{\mathbf{A}}(m) \cdot \gamma_m^{-1}$. Equating the left- and right-hand sides of Equation S42 with the constant of Equation S51 gives

$$\langle \overleftarrow{q} \rangle_m = \frac{1}{\gamma_m} \left[\frac{\langle \alpha_n \beta_n^{-1} \rangle}{\langle \beta_n^{-1} \rangle + 2MN^{-1} \langle \gamma_m^{-1} \rangle} \right] \tag{S52}$$

and

$$\langle \overrightarrow{q} \rangle_n = \frac{1}{2\beta_n} \left[\alpha_n - \frac{\langle \alpha_n \beta_n^{-1} \rangle}{\langle \beta_n^{-1} \rangle + 2MN^{-1} \langle \gamma_m^{-1} \rangle} \right], \tag{S53}$$

respectively. By Equation S53, in the case that $\alpha_n \equiv \alpha_0$ (as in all cases considered in the article), efficient states are thus characterized by uniform expected source quality $\langle b \rangle_n = \alpha_0/2 - K$, and are thus also steady states of the reallocation dynamics of Equation S3. Similarly, by Equation S52, if $\gamma_m \equiv \gamma_0$ (as in all cases considered in the article), these states are also characterized by degree-independent levels of total agent extraction $\langle \overleftarrow{q} \rangle_m = K/\gamma_0$ and fitness

$$\langle f \rangle_m = \frac{K}{\gamma_0} \left(\frac{\alpha_0}{2} - K \right). \tag{S54}$$

Total collective extraction $Q = M \langle \overleftarrow{q} \rangle_m$ and wealth $F = M \langle f \rangle_m$ can then be computed using Equations S52 and S54, respectively. This expression for collective wealth of efficient states (labelled as F_{Ef}) is used to compute the estimates of efficiency shown Figure 2a-b and Figure S1a-b.

S4 Results: degree-proportional capacity scenario

The results presented in the main article represent a *uniform capacity* scenario in which a source's quality is assumed to degrade in proportion to the overall extraction pressure it receives regardless of its number of affiliated users. Here, we present the counterparts of those results that instead represent a *degree-proportional capacity* scenario, where CPRs are assumed to degrade in proportion to the amount of extraction pressure *per affiliated agent* that they receive. *Degree-proportional capacity* counterparts for Figure 2, 3, and 4 are displayed below in Figure S1, S2, and S3, respectively. We find that the basic conclusions drawn for the *uniform capacity* scenario are robust with respect to this change of dependence of CPR capacity on degree.

A key difference in the *degree-proportional capacity* scenario is that agents come to exert a greater relative proportion of their extraction effort towards higher-degree sources at equilibrium than in the *uniform capacity* scenario. This occurs because higher-degree sources are degraded to a lesser extent (and lower-degree sources are degraded to a greater extent) by each additional unit of extraction pressure here than they are in the *uniform capacity* scenario, which motivates rational agents to reply more on higher-degree sources and less on lower-degree sources when $\beta_n \propto n^{-1}$ than they would if β_n were constant (see Equations S17 and S28). Since higher-degree sources suffer greater degradation at equilibrium than do lower-degree sources, networks that contain very large-degree (low-quality) "hub" sources tend to operate less efficiently at equilibrium in the degree-proportional capacity scenario. This stands in contrast to the uniform capacity scenario, in which networks with greater source degree heterogeneity tend to operate *more* efficiently at equilbrium. However, despite this difference, networks with higher source degree heterogeneity still experience greater *increases* in collective wealth as a result of reallocation dynamics from equilbrium in this *degree-proportional capacity* scenario, again for the same reasons as in the *uniform capacity* case.

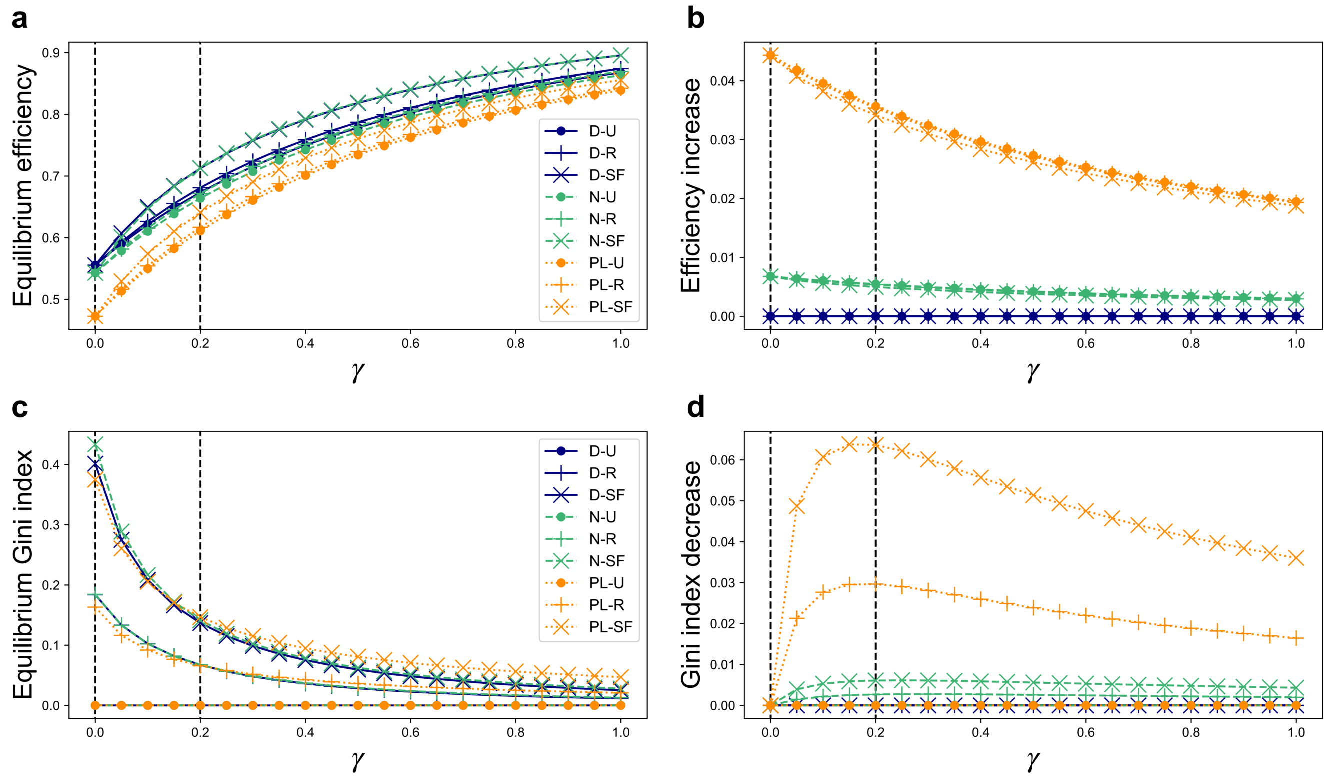

Figure S1. Estimates of (a) Ratio of total collective wealth of equilibrium ("Eq") states relative to efficient ("Ef") states, $F_{\text{Eq}}/F_{\text{Ef}}$; (b) increase in efficiency from equilibrium to steady states ("SS"), $(F_{\text{SS}} - F_{\text{Eq}})/F_{\text{Ef}}$; (c) Gini index of equilibrium states G_{Eq}; and (d) decrease in Gini index from equilibrium to steady states, $(G_{\text{Eq}} - G_{\text{SS}})$, all as functions of cost parameter γ. Results shown correspond to a *degree-proportional capacity* scenario with $\alpha_0 = \beta_0 = 1$.

S5 Computing extraction states on networks

S5.1 Computing steady states of reallocation dynamics

For steady states of the dynamics described by Equation S3, the condition $\frac{\mathrm{d}}{\mathrm{d}t}q(a,s) = 0$ for all $q(a,s)$ requires that all sources affiliated with each agent a share a common quality value:

$$\sum_{s' \in \mathbf{S}_a \setminus \{s\}} \left[\left(\alpha(s) - \beta(s) \sum_{a' \in \mathbf{A}_s} q(a',s) \right) - \left(\alpha(s') - \beta(s') \sum_{a'' \in \mathbf{A}_{s'}} q(a'',s') \right) \right] \tag{S55}$$

$$= (m(a)-1) \left(\alpha(s) - \beta(s) \sum_{a' \in \mathbf{A}_s} q(a',s) \right) - \sum_{s' \in \mathbf{S}_a \setminus \{s\}} \left(\alpha(s') - \beta(s') \sum_{a'' \in \mathbf{A}_{s'}} q(a'',s') \right) = 0. \tag{S56}$$

We denote as $\tilde{\mathbf{Q}}$ the vector of extraction effort values $q(a,s)$ for all edges $(a,s) \in \mathbf{L}$ of the network at hand that involve agents with $m(a) > 1$. Indexing these entries such that $Q_{(a,s)} = q(a,s)$, we express the linear system above as

$$\mathbf{C}_{\text{SS}} \tilde{\mathbf{Q}} \geq \vec{\Delta\alpha} + \tilde{\mathbf{x}} \tag{S57}$$

with entries of the matrix $\mathbf{C}_{\text{SS}}$ given by

$$(C_{\text{SS}})_{(a,s),(a',s')} = \begin{cases} -(m(a)-1)\beta(s), & \text{if } s = s' \\ \beta(s') & \text{if } s' \in \mathbf{S}_a \setminus \{s\} \\ 0 & \text{otherwise} \end{cases}, \tag{S58}$$

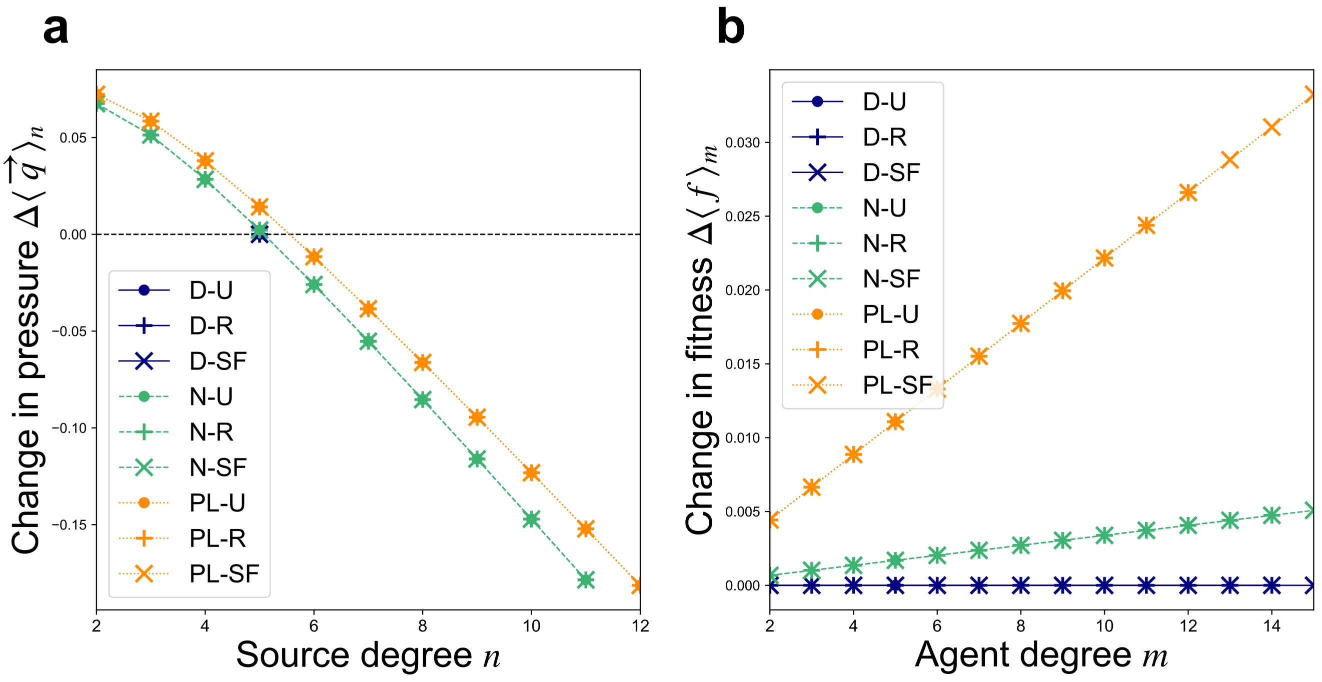

Figure S2. Estimated shifts in extraction patterns due to reallocation dynamics from Nash equilibrium ("Eq") to steady states ("SS") under cost-free extraction: (a) Change in total extraction pressure $\Delta\langle\vec{q}\rangle_n = \langle\vec{q}\rangle_{n,\mathrm{SS}} - \langle\vec{q}\rangle_{n,\mathrm{Eq}}$, as a function of source degree n; and (b) change in expected agent fitness, $\Delta\langle f\rangle_m = \langle f\rangle_{m,\mathrm{SS}} - \langle f\rangle_{m,\mathrm{Eq}}$ as a function of agent degree m. Results shown correspond to a *degree-proportional capacity* scenario with $\alpha_0 = \beta_0 = 1$ and $\gamma_0 = 0$. Note that results for all network types sharing a common source degree distribution type ("D", "N", or "PL") are overlapping.

and where on the right-hand side of Equation S57, $\vec{\Delta\alpha}$ is given by

$$\vec{\Delta\alpha} = (m(a)-1)\alpha(s) - \sum_{s'\in\mathbf{S}_a\setminus\{a\}} \alpha(s'), \tag{S59}$$

and $\tilde{\mathbf{x}}$ accounts for extraction by agents that have $m(a') = 1$ and are thus incapable of reallocating extraction:

$$\tilde{\mathbf{x}}_{(a,s)} = (m(a)-1)\beta(s)\left(\sum_{a'\in\mathbf{A}_s} q(a',s)\cdot\delta_{m(a'),1}\right) - \sum_{s'\in\mathbf{S}_a\setminus\{s\}} \beta(s')\left(\sum_{a''\in\mathbf{A}_s} q(a'',s')\cdot\delta_{m(a''),1}\right), \tag{S60}$$

where $\delta_{i,j} = 1$ if $i = j$ and $\delta_{i,j} = 0$ if $i \neq j$. The system of Equation S57 along with the constraint

$$\tilde{\mathbf{Q}} \geq 0, \tag{S61}$$

represent a linear complementarity problem. We solve this numerically using quadratic programming algorithms (here, we use Python 3.7.3 with SciPy 1.2.1[4]) that minimize the objective function

$$J\left(\tilde{\mathbf{Q}}\right) = \tilde{\mathbf{Q}}^T\left(\mathbf{C}_{\mathrm{SS}}\tilde{\mathbf{Q}} - \left(\vec{\Delta\alpha} + \tilde{\mathbf{x}}\right)\right) \tag{S62}$$

subject to the constraints of Equation S57 and Equation S61. We impose the further constraint that agents' extraction intensity values remain equal to those of the initial condition:

$$\mathbf{R}\tilde{\mathbf{Q}} = \tilde{\mathbf{T}}, \tag{S63}$$

where $\mathbf{R}$ is a matrix with a row for each agent a having $m(a) > 1$, and a column for each edge (a,s), and entries

$$R_{a,(a',s')} = \begin{cases} 1 & \text{if } a = a' \\ 0 & \text{if } a \neq a' \end{cases}, \tag{S64}$$

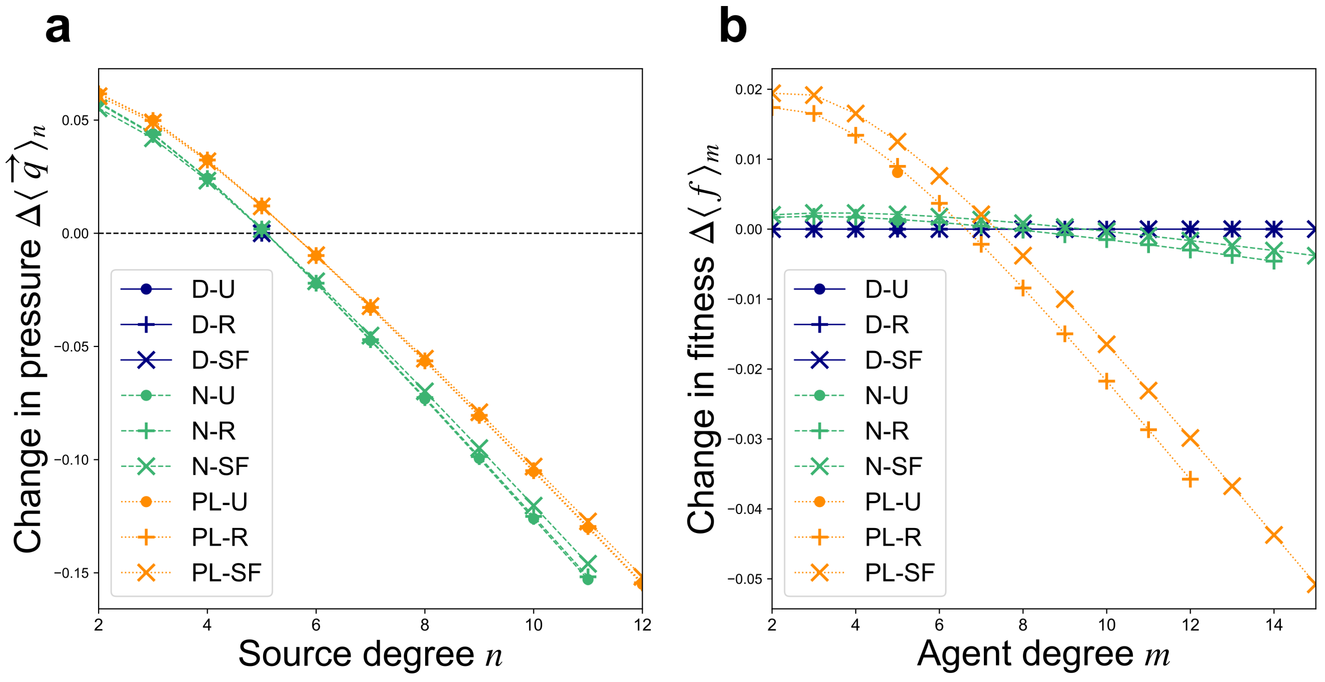

Figure S3. Estimated shifts in extraction patterns due to reallocation dynamics from Nash equilibrium ("Eq") to steady states ("SS") under costly extraction: (a) Change in total extraction pressure $\Delta\langle\overrightarrow{q}\rangle_n = \langle\overrightarrow{q}\rangle_{n,\text{SS}} - \langle\overrightarrow{q}\rangle_{n,\text{Eq}}$, as a function of source degree n; and (b) change in expected agent fitness, $\Delta\langle f\rangle_m = \langle f\rangle_{m,\text{SS}} - \langle f\rangle_{m,\text{Eq}}$ as a function of agent degree m. Results shown correspond to a *degree-proportional capacity* scenario with $\alpha_0 = \beta_0 = 1$ and $\gamma_0 = 0.2$.

and the vector $\tilde{\mathbf{T}}$ has entries equal to the initial extraction intensity values $\overleftarrow{q_0}(a)$ for each agent a that has $m(a) > 1$:

$$T_a = \overleftarrow{q_0}(a). \tag{S65}$$

For the results presented in Section S6, where the initial conditions are Nash equilibrium extraction states, these values correspond to the Nash equilibrium values of extraction intensity computed as detailed below in Section S5.2. To provide an initial guess to the algorithm, we use a forward Euler numerical scheme to advance Nash equilibrium states (computed as described below in Section S5.2) in time under Equation S3 with $k = 10^{-3}$ for 10^3 iterations. Averaging over the resulting steady states for each network ensemble, degree-binned values of mean extraction pressure $\overrightarrow{q}(s)$ and mean agent extraction intensity $\overleftarrow{q}(a)$ along with corresponding values for Nash equilibrium states, are shown for representative cases of cost-free and costly extraction in Figures S4, S5, S6, S7, S8, S9, S10, and S11. Results for collective wealth and Gini indices of these steady states of reallocation dynamics are shown in Tables S1, S2, S3, and S4.

In addition to its use in computing the steady states themselves, the eigenvalues of the matrix $\mathbf{C}_{\text{SS}}$ can also be used to study the stability of these fixed points. The reallocation dynamics of Equation S3 are equivalent to

$$\frac{\mathrm{d}}{\mathrm{d}t}\tilde{\mathbf{Q}} = k\mathbf{C}_{\text{SS}}\tilde{\mathbf{Q}}. \tag{S66}$$

Referring to Equation S58, the trace of $\mathbf{C}_{\text{SS}}$, equal to the sum of its eigenvalues, is

$$\text{Tr}\left(\tilde{\mathbf{Q}}_{\text{SS}}\right) = -\sum_{(a,s)\in\mathbf{L}} (m(a)-1)\,\beta(s) \tag{S67}$$

$$= -M\sum_{m=1}^{m_{\max}} P_{\mathbf{A}}(m)\cdot\left[m(m-1)\left(\sum_{n=1}^{n_{\max}} P_{\mathbf{S}}(n\mid m)\cdot\beta_n\right)\right] = -M\sum_{m=1}^{m_{\max}} P_{\mathbf{A}}(m)\cdot\left[m(m-1)\langle\beta_n\rangle_m\right] \tag{S68}$$

$$= -\frac{M\langle n\beta_n\rangle}{\langle n\rangle}\sum_{m=1}^{m_{\max}} P_{\mathbf{A}}(m)\cdot\left[m(m-1)\right] = -\frac{M\langle n\beta_n\rangle}{\langle n\rangle}\left[\langle m^2\rangle - \langle m\rangle\right], \tag{S69}$$

where in the final line we have assumed an absence of degree-degree correlations. When comparing this value for networks with shared values of M and $\langle m\rangle$, the sum of the eigenvalues is then larger for networks with larger values of $\langle m^2\rangle$, that is,

with higher variance among agent degrees. So, greater agent degree heterogeneity is associated with negative eigenvalues of greater magnitude, and so with faster rates of convergence towards steady states under reallocation dynamics. The presence of higher-degree agents, who serve to simultaneously distribute extraction pressure between their many affiliated sources, helps quality values among sources to become more quickly equalized. For both the *uniform capacity* and *degree-proportional capacity* scenarios considered in the article, $\mathrm{Tr}\left(\mathbf{C}_{\mathrm{SS}}\right) = M\beta_0\left[\langle m^2\rangle - \langle m\rangle\right]$, and so variability among CPR source degrees does not affect the sum of these eigenvalues.

S5.2 Computing Nash equilibrium states

To compute the Nash equilibrium extraction state for a given network, we follow İlkılıç[2], but generalize to include possible variability among parameter values for different sources and agents. Using Equation S1, the Nash equilibrium (best-response) extraction levels $q(a,s) > 0$ are given by the zeroes of

$$\frac{\partial f(a)}{\partial q(a,s)} = b(s) + \frac{\partial b(s)}{\partial q(a,s)} q(a,s) - \gamma(a)\left(\sum_{s' \in \mathbf{S}_a} q(a,s')\right), \tag{S70}$$

and since by Equation S2, $\frac{\partial b(s)}{\partial q(a,s)} = -\beta(s)$, we have

$$q(a,s) = \left(\frac{1}{2\beta(s)+\gamma(a)}\right)\left[\alpha(s) - \beta(s)\left(\sum_{a' \in \mathbf{A}_s \setminus \{a\}} q(a',s)\right) - \gamma(a)\left(\sum_{s' \in \mathbf{S}_a \setminus \{s\}}\right)\right]. \tag{S71}$$

Expressing this system of conditions in matrix form, we have

$$\left[\mathbf{I} - \mathbf{X}\mathbf{C}_{\mathrm{Eq}}\right]\tilde{\mathbf{Q}} \geq \mathbf{X}\tilde{\alpha}, \tag{S72}$$

where $\mathbf{X}$ has entries

$$X_{(a,s),(a',s')} = \begin{cases} (2\beta(s)+\gamma(a))^{-1} & \text{if } a = a' \text{ and } s \neq s' \\ 0 & \text{otherwise} \end{cases}, \tag{S73}$$

the vector $\tilde{\alpha}$ has entries $\vec{\alpha}_{(a,s)} = \alpha(s)$, and the matrix $\mathbf{C}_{\mathrm{Eq}}$ has entries

$$\left(C_{\mathrm{Eq}}\right)_{(a,s),(a',s')} = \begin{cases} \gamma(a) & \text{if } a = a' \text{ and } s \neq s' \\ \beta(s) & \text{if } a \neq a' \text{ and } s = s' \\ 0 & \text{otherwise} \end{cases}. \tag{S74}$$

This generalizes the previous results of İlkılıç[2] to allow for individual variations in the values of the parameters $\alpha(s)$ and $\beta(s)$ among CPR sources and in the values of $\gamma(a)$ among agents.

As were the conditions for steady states, this presents a linear complementarity problem that we solve numerically by using quadratic programming algorithm to minimize the objective function

$$J\left(\tilde{\mathbf{Q}}\right) = \tilde{\mathbf{Q}}^T\left(\left[\mathbf{I} - \mathbf{X}\mathbf{C}_{\mathrm{Eq}}\right]\tilde{\mathbf{Q}} - \mathbf{X}\tilde{\alpha}\right) \tag{S75}$$

subject to the constraints of Equation S72 and the requirement that $\tilde{\mathbf{Q}} \geq 0$. Degree-binned values of mean extraction pressure $\overrightarrow{q}(s)$ and mean agent extraction intensity $\overleftarrow{q}(a)$ averaged over these Nash equilibrium states for each network ensemble, along with corresponding values for evolutionary steady states, are shown in Figure S4, S5, S6, S7, S8, S9, S10, and S11.

S5.3 Computing Pareto efficient states

To compute efficient states, we again follow and generalize from the work of İlkılıç, but approach the computation as a linear complementarity problem rather than construct solutions based on "least inclusive subgraphs"[2]. Efficient extraction states are those that maximize the population's total utility, which by the payoff function of Equation S1 is

$$F = \sum_{a \in \mathbf{A}} f(a) = \sum_{a \in \mathbf{A}}\left[\left(\sum_{s \in \mathbf{S}_a} q(a,s) \cdot b(s)\right) - \frac{\gamma(a)}{2}\left(\sum_{s \in \mathbf{S}_a} q(a,s)\right)^2\right]. \tag{S76}$$

The condition

$$\frac{\partial F}{\partial q(a,s)} = b(s) + \frac{\partial b(s)}{\partial q(a,s)} \left(\sum_{a' \in \mathbf{A}_s} q(a,s') \right) - \gamma(a) \left(\sum_{s' \in \mathbf{S}_a} q(a,s') \right) = 0, \tag{S77}$$

with Equation S2 and $\frac{\partial b(s)}{\partial q(a,s)} = -\beta(s)$ becomes

$$2\beta(s) \left(\sum_{a' \in \mathbf{A}_s} q(a,s') \right) + \gamma(a) \left(\sum_{s' \in \mathbf{S}_a} q(a,s') \right) = \alpha(s). \tag{S78}$$

In matrix form, the system of conditions on all edges $(a,s) \in \mathbf{L}$ of Equation S78 is

$$\mathbf{C}_{\mathrm{Ef}} \tilde{\mathbf{Q}} \geq \tilde{\alpha}, \tag{S79}$$

where the entries of $\mathbf{C}_{\mathrm{Ef}}$ are

$$(C_{\mathrm{Ef}})_{(a,s),(a',s')} = \begin{cases} \gamma(a) & \text{if } a = a' \text{ and } s \neq s' \\ 2\beta(s) & \text{if } a \neq a' \text{ and } s = s' \\ 0 & \text{otherwise} \end{cases}, \tag{S80}$$

and $\tilde{\alpha}_{(a,s)} = \alpha(s)$. We solve this linear complementarity problem using quadratic programming algorithms to minimize the objective function

$$J\left(\tilde{\mathbf{Q}}\right) = \tilde{\mathbf{Q}}^T \left(\mathbf{C}_{\mathrm{Ef}} \tilde{\mathbf{Q}} - \tilde{\alpha} \right) \tag{S81}$$

subject to the constraint of Equation S79 and $\tilde{\mathbf{Q}} \geq 0$. As indicated in previous literature[2], we note that the particular solution thus obtained may not necessarily represent the only possible efficient state of a given network. The resulting efficient extraction states are used to compute the efficiency values (i.e. the collective wealth of an extraction state expressed as a fraction of its maximum possible value, which is reached in Pareto efficient states) shown in Tables S1, S2, S3, and S4.

S6 Validation

We now aim to validate the estimates for the degree dependence of source extraction pressure and agent fitness which were computed using ensemble degree distributions as detailed above in Section S3. Nash equilibrium, Pareto efficient, and steady states of reallocation dynamics are numerically computed as described in Section S5 for 10^3 individual network realizations of each of the 9 network types whose ensemble-mean degree distributions are shown in Figure 1. Degree-binned mean values of these quantities, averaged over each of the ensembles, are plotted separately for random ("R") and scale-free-type ("SF") agent degree distributions. For the *uniform capacity* scenario, results for cost-free extraction ($\gamma_0 = 0$) are shown in Figure S4 ("R") and Figure S5 ("SF"), corresponding to the estimates shown in Figure 3. Results for a representative case of costly extraction ($\gamma_0 = 0.2$) are shown in Figure S6 ("R") and Figure S7 ("SF"), corresponding to the estimates shown in Figure 4. Results for the *degree-proportional capacity* scenario are shown for cost-free extraction ($\gamma_0 = 0$) in Figure S8 ("R") and Figure S9 ("SF"), corresponding to the estimates shown in Figure S2, and for a representative case of costly extraction ($\gamma_0 = 0.2$) in Figure S10 ("R") and Figure S11 ("SF"), corresponding to the estimates shown in Figure S3.

The observed ensemble-mean shifts in extraction pressure and agent fitness tend to show strong agreement with the heterogeneous mean-field predictions, particularly in terms of the qualitative trends they exhibit. In several cases, the features of the degree dependence predicted closely resemble that observed in ensemble means, including the relative differences between the curves representing different network types, but with the observed mean shifts being far more exaggerated than those predicted by the model (e.g. Figures S4, S7, and S11). An interesting and consistent qualitative deviation of ensemble mean results from predictions is seen across networks with random ("R") degree distributions, where the relatively small class of lowest-degree ($m(a) \leq 3$) agents actually tend to have their fitnesses *reduced* by reallocation dynamics in the cost-free case (Figures S4 and S8), or experience fitness increases much smaller than those predicted in the case of costly extraction (Figures S6 and S10). This suggests that the approximations used in these estimates – somewhat similar in approach to other heterogeneous mean-field models of network games[5] – seem to break down for smaller-degree agents, whose smaller "sample sizes" of sources lead them to vary further from the statistical predictions especially in the presence of large source degree heterogeneity. Predictions disagree most drastically with observations for the case of cost-free extraction on networks with high degree heterogeneity in both source and agent degrees ("PL-SF"), where the observed fitness shifts decrease with agent

degree as the predicted shifts increase linearly (Figures S5 and S9). By treating all sources of a common degree in terms of a single mean quantity, the approach fails to distinguish discrepancies in quality among sources of a common degree under costly extraction, and so fails to predict the increases in wealth and equality observed in the case of costly extraction even on networks with delta-function source degree distributions ("D") where all sources share a common degree, $n(s) \equiv \langle n \rangle$; (Figures S6, S7, S10, and S11). On actual networks, the fitness shifts observed on these "D" networks resemble those of networks with normal ("N") source degree distributions.

At the population level, ensemble mean collective wealth (expressed as ratios of the efficient-state collective wealth) and Gini index values, along with their changes under reallocation dynamics, are provided in Table S1, S2, S3, and S4. These Gini index values can be compared with the corresponding estimated Gini indices for $\gamma_0 = 0$ and $\gamma_0 = 0.2$ highlighted by vertical lines in Figure 2 and Figure S1. Gini indices of each extraction state $\tilde{\mathbf{Q}}$ are computed as

$$G\left(\tilde{\mathbf{Q}}\right) = \frac{1}{2M^2\mu} \sum_{a_1 \in \mathbf{A}} \sum_{a_2 \in \mathbf{A}} |f(a_1) - f(a_2)|, \tag{S82}$$

where $\mu = \left(\sum_{a \in \mathbf{A}} f(a)\right)/M$, with payoffs $f(a)$ computed according to Equation S1 using the values $q(a,s) = Q_{(a,s)}$. Observed efficiency values and Gini indices show strong quantitative agreement with predictions, as do shifts in efficiency that result from reallocation dynamics. The heterogeneous mean-field approach, by ignoring higher-order types of diversity among agents beyond their degrees, tends to greatly underestimate the observed shifts in Gini index; even networks with delta-function ("D") source degree distributions show increased equality under costly extraction (Tables S2 and S4) due to differences among sources of common degree which the model is incapable of capturing. Despite these sources of quantitative differences between predictions and ensemble-mean observations in certain cases, computations on actual networks nonetheless consistently confirm that myopic reallocations tend to increase both collective wealth and wealth equality in this CPR extraction game.

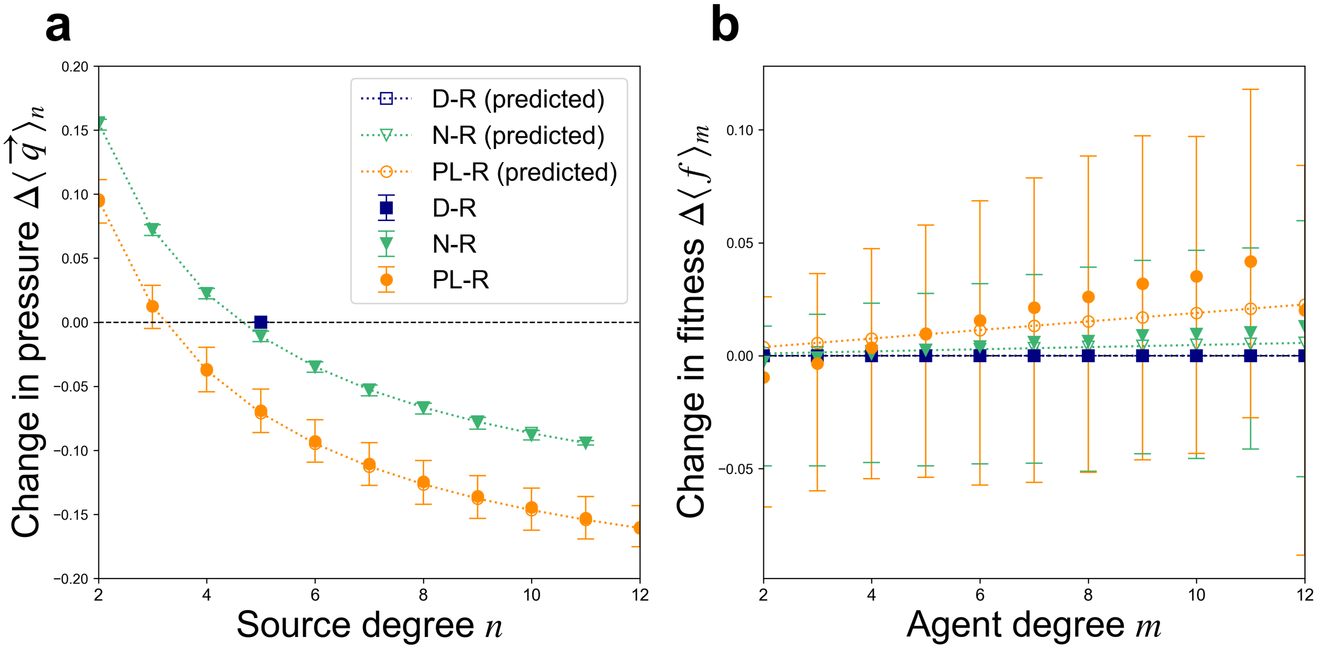

Figure S4. Mean changes in Nash equilibrium extraction patterns under reallocation dynamics with cost-free extraction (Random agent degree distributions): (a) Change in total extraction pressure $\Delta\langle\vec{q}\rangle_n = \langle\vec{q}\rangle_{n,\mathrm{SS}} - \langle\vec{q}\rangle_{n,\mathrm{Eq}}$, as a function of source degree n; and (b) change in expected agent fitness $\Delta\langle f\rangle_m = \langle f\rangle_{m,\mathrm{SS}} - \langle f\rangle_{m,\mathrm{Eq}}$ as a function of agent degree m for the *uniform capacity* scenario ($\alpha_0 = \beta_0 = 1$, $\gamma_0 = 0$). Lower and upper error bars indicate the 2.5th and 97.5th percentiles, respectively.

References

1. Ohkubo, J., Tanaka, K. & Horiguchi, T. Generation of complex bipartite graphs by using a preferential rewiring process. *Physical Review E* **72**, 036120 (2005).

2. İlkılıç, R. Networks of common property resources. *Economic Theory* **47**, 105–134 (2011).

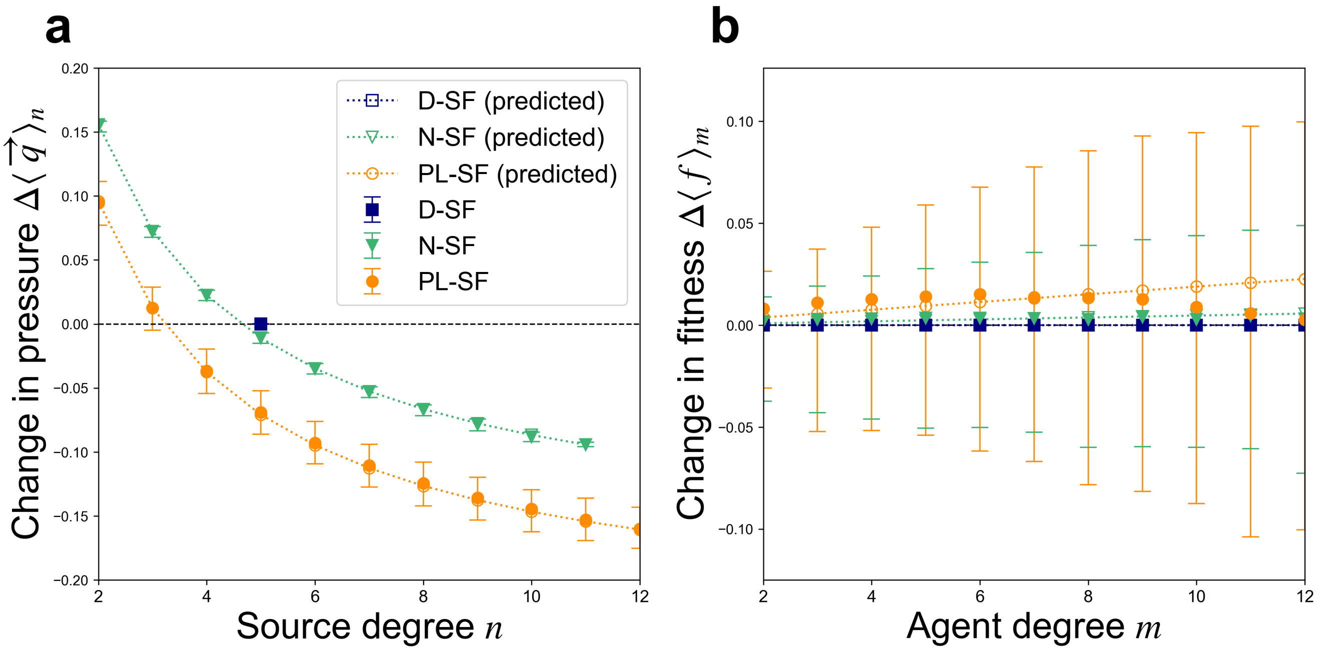

Figure S5. Mean changes in Nash equilibrium extraction patterns under reallocation dynamics with cost-free extraction (Scale-free-type agent degree distributions): (a) Change in total extraction pressure $\Delta\langle\vec{q}\rangle_n = \langle\vec{q}\rangle_{n,\mathrm{SS}} - \langle\vec{q}\rangle_{n,\mathrm{Eq}}$, as a function of source degree n; and (b) change in expected agent fitness $\Delta\langle f\rangle_m = \langle f\rangle_{m,\mathrm{SS}} - \langle f\rangle_{m,\mathrm{Eq}}$ as a function of agent degree m for the *uniform capacity* scenario ($\alpha_0 = \beta_0 = 1$, $\gamma_0 = 0$). Lower and upper error bars indicate the 2.5th and 97.5th percentiles, respectively.

3. Mukhopadhyay, N. *Probability and statistical inference* (CRC Press, 2000).

4. Virtanen, P. *et al.* SciPy 1.0: Fundamental Algorithms for Scientific Computing in Python. *Nature Methods* **17**, 261–272 (2020).

5. Cimini, G. Evolutionary network games: Equilibria from imitation and best response dynamics. *Complexity* **2017** (2017).

Network type	Equilibrium efficiency (%)	Equilibrium Gini index	Efficiency increase (%)	Gini index decrease
D-U	55.6	0	0	0
D-R	55.6	.182	0	0
D-SF	55.6	.398	0	0
N-U	57.5	.129	0.9	.070
N-R	57.5	.215	0.9	.026
N-SF	57.5	.469	0.9	.023
PL-U	68.7	.246	3.8	.10
PL-R	68.7	.261	3.8	.07
PL-SF	68.7	.532	3.8	.06

Table S1. Ensemble mean values of equilibrium efficiency $F_{\mathrm{Eq}}/F_{\mathrm{Ef}}$, Gini index G_{Eq}, change in efficiency $(F_{\mathrm{SS}} - F_{\mathrm{Eq}})/F_{\mathrm{Ef}}$, and decrease in Gini index $(G_{\mathrm{SS}} - G_{\mathrm{Eq}})$ for 9 network types. Results correspond to a *uniform capacity* scenario with cost-free extraction ($\alpha_0 = \beta_0 = 1$, $\gamma_0 = 0$).

Network type	Equilibrium efficiency (%)	Equilibrium Gini index	Efficiency increase (%)	Gini index decrease
D-U	67.4	0	.0	0
D-R	68.1	.086	0.0	.013
D-SF	71.1	.176	0.2	.027
N-U	68.5	.079	0.9	.056
N-R	69.2	.114	0.9	.037
N-SF	72.9	.219	1.1	.054
PL-U	75.9	.170	3.6	.109
PL-R	76.3	.174	3.4	.090
PL-SF	77.4	.353	4.3	.127

Table S2. Ensemble mean values of equilibrium efficiency $F_{\mathrm{Eq}}/F_{\mathrm{Ef}}$, Gini index G_{Eq}, change in efficiency $(F_{\mathrm{SS}} - F_{\mathrm{Eq}})/F_{\mathrm{Ef}}$, and decrease in Gini index $(G_{\mathrm{SS}} - G_{\mathrm{Eq}})$ for 9 network types. Results correspond to a *uniform capacity* scenario with costly extraction ($\alpha_0 = \beta_0 = 1$, $\gamma_0 = 0.2$).

Network type	Equilibrium efficiency (%)	Equilibrium Gini index	Efficiency increase (%)	Gini index decrease
D-U	55.6	0	0	0
D-R	55.6	.182	0	0
D-SF	55.6	.398	0	0
N-U	54.3	.044	0.7	.033
N-R	54.3	.186	0.7	.000
N-SF	54.3	.442	0.7	.011
PL-U	47.3	.114	4.4	.088
PL-R	47.3	.181	4.4	.011
PL-SF	47.3	.455	4.4	.091

Table S3. Ensemble mean values of equilibrium efficiency $F_{\mathrm{Eq}}/F_{\mathrm{Ef}}$, Gini index G_{Eq}, change in efficiency $(F_{\mathrm{SS}} - F_{\mathrm{Eq}})/F_{\mathrm{Ef}}$, and decrease in Gini index $(G_{\mathrm{SS}} - G_{\mathrm{Eq}})$ for 9 network types. Results correspond to a *degree-proportional capacity* scenario with cost-free extraction ($\alpha_0 = \beta_0 = 1$, $\gamma_0 = 0$).

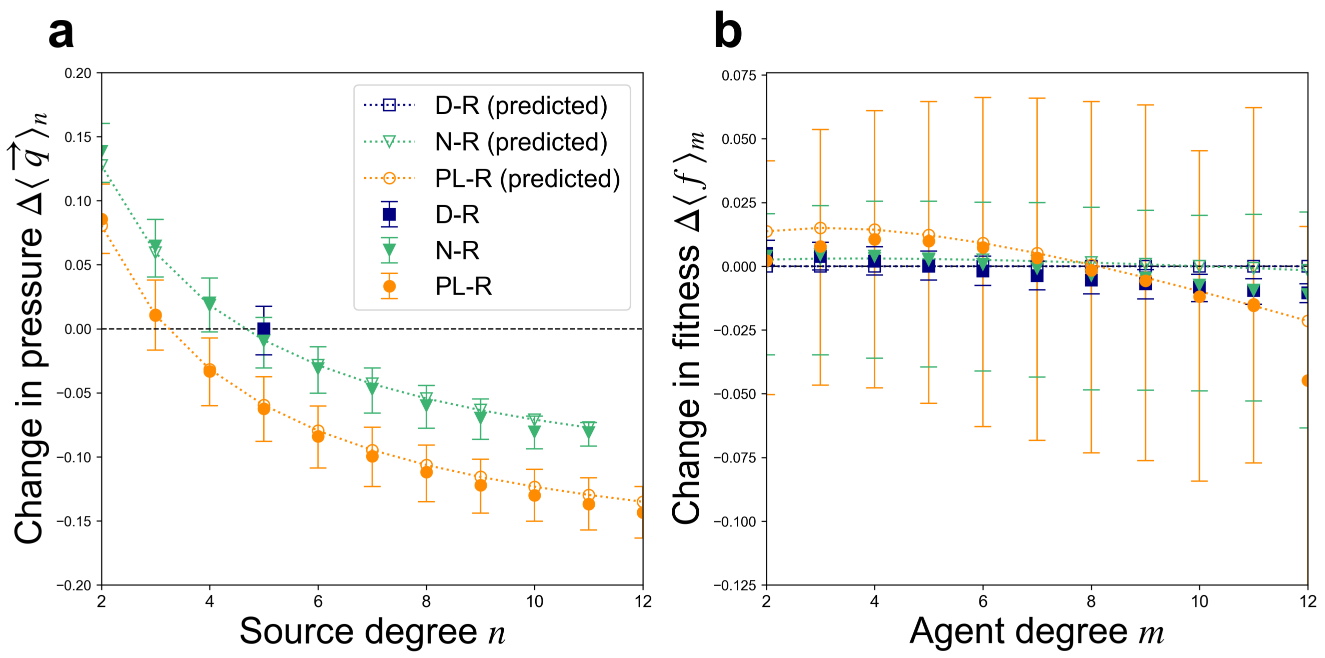

Figure S6. Mean changes in Nash equilibrium extraction patterns under reallocation dynamics with costly extraction (Random agent degree distributions): (a) Change in total extraction pressure $\Delta\langle\vec{q}\rangle_n = \langle\vec{q}\rangle_{n,\mathrm{SS}} - \langle\vec{q}\rangle_{n,\mathrm{Eq}}$, as a function of source degree n; and (b) change in expected agent fitness $\Delta\langle f\rangle_m = \langle f\rangle_{m,\mathrm{SS}} - \langle f\rangle_{m,\mathrm{Eq}}$ as a function of agent degree m for the *uniform capacity* scenario ($\alpha_0 = \beta_0 = 1$, $\gamma_0 = 0$). Lower and upper error bars indicate the 2.5th and 97.5th percentiles, respectively.

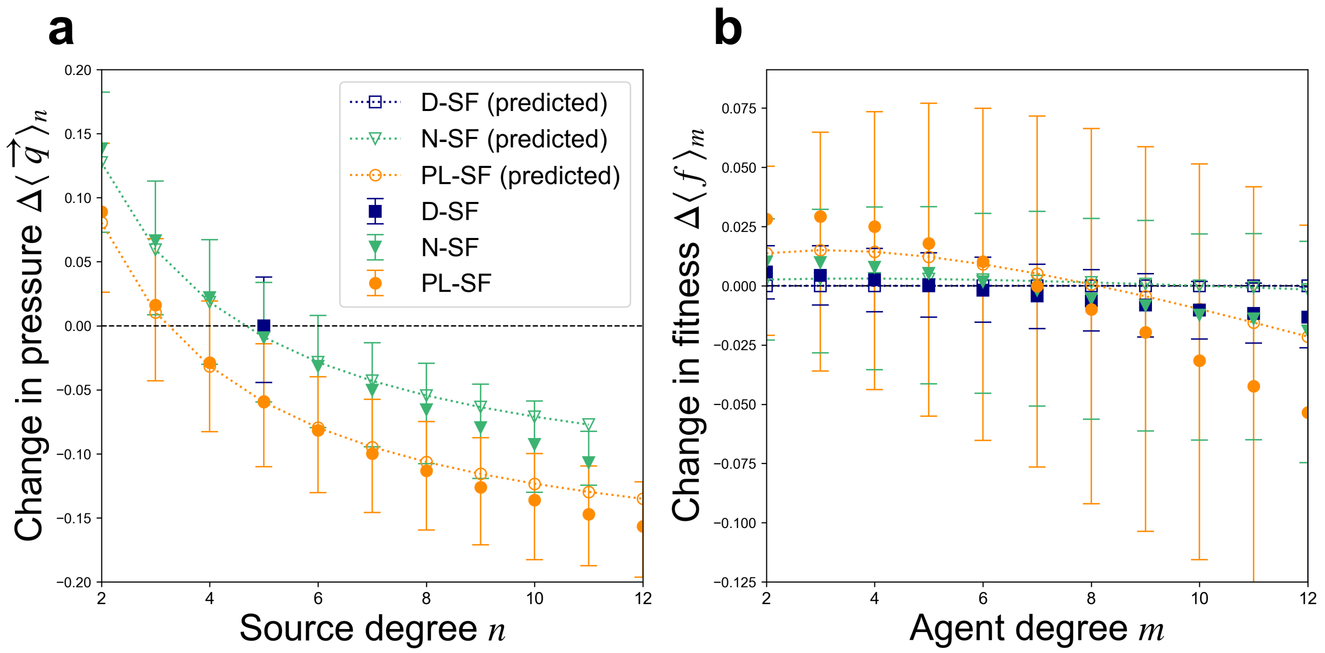

Figure S7. Mean changes in Nash equilibrium extraction patterns under reallocation dynamics with costly extraction (Scale-free-type agent degree distributions): (a) Change in total extraction pressure $\Delta\langle\vec{q}\rangle_n = \langle\vec{q}\rangle_{n,\mathrm{SS}} - \langle\vec{q}\rangle_{n,\mathrm{Eq}}$, as a function of source degree n; and (b) change in expected agent fitness $\Delta\langle f\rangle_m = \langle f\rangle_{m,\mathrm{SS}} - \langle f\rangle_{m,\mathrm{Eq}}$ as a function of agent degree m for the *uniform capacity* scenario ($\alpha_0 = \beta_0 = 1$, $\gamma_0 = 0$). Lower and upper error bars indicate the 2.5th and 97.5th percentiles, respectively.

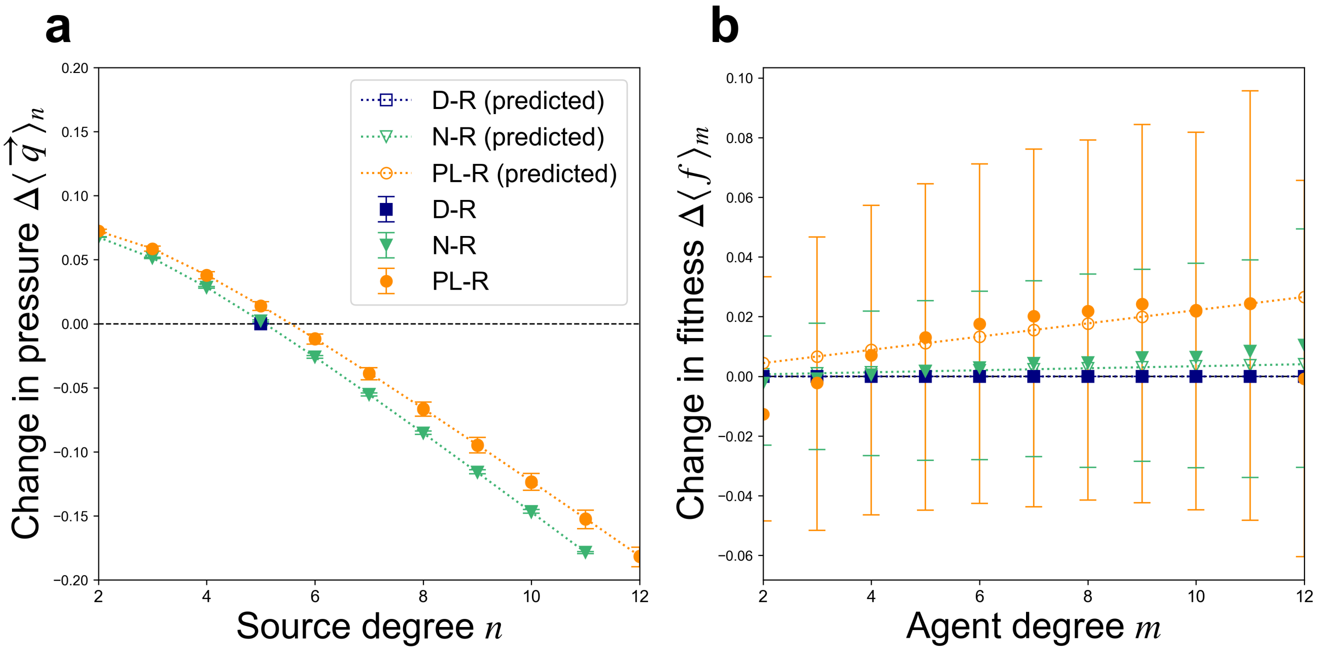

Figure S8. Mean changes in Nash equilibrium extraction patterns under reallocation dynamics with cost-free extraction (Random agent degree distributions): (a) Change in total extraction pressure $\Delta\langle\overrightarrow{q}\rangle_n = \langle\overrightarrow{q}\rangle_{n,\mathrm{SS}} - \langle\overrightarrow{q}\rangle_{n,\mathrm{Eq}}$, as a function of source degree n; and (b) change in expected agent fitness $\Delta\langle f\rangle_m = \langle f\rangle_{m,\mathrm{SS}} - \langle f\rangle_{m,\mathrm{Eq}}$ as a function of agent degree m for the *degree-proportional capacity* scenario ($\alpha_0 = \beta_0 = 1$, $\gamma_0 = 0$). Lower and upper error bars indicate the 2.5th and 97.5th percentiles, respectively.

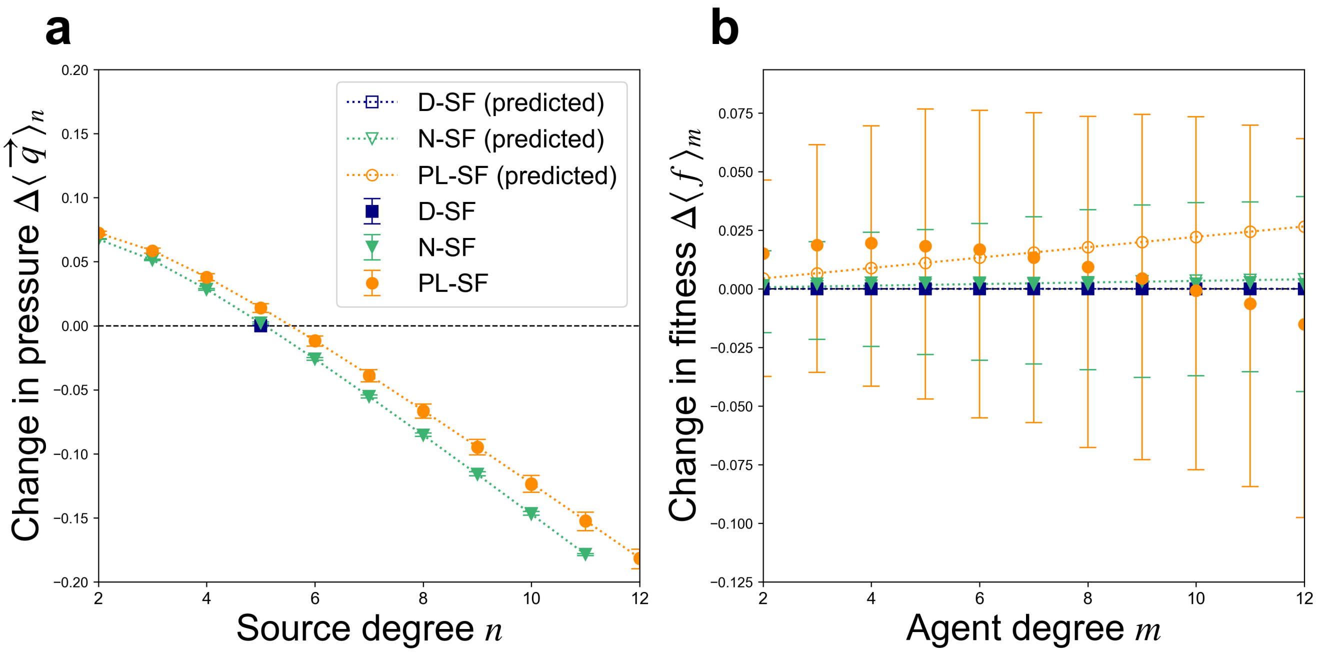

Figure S9. Mean changes in Nash equilibrium extraction patterns under reallocation dynamics with cost-free extraction (Scale-free-type agent degree distributions): (a) Change in total extraction pressure $\Delta\langle\overrightarrow{q}\rangle_n = \langle\overrightarrow{q}\rangle_{n,\mathrm{SS}} - \langle\overrightarrow{q}\rangle_{n,\mathrm{Eq}}$, as a function of source degree n; and (b) change in expected agent fitness $\Delta\langle f\rangle_m = \langle f\rangle_{m,\mathrm{SS}} - \langle f\rangle_{m,\mathrm{Eq}}$ as a function of agent degree m for the *degree-proportional capacity* scenario ($\alpha_0 = \beta_0 = 1$, $\gamma_0 = 0$). Lower and upper error bars indicate the 2.5th and 97.5th percentiles, respectively.

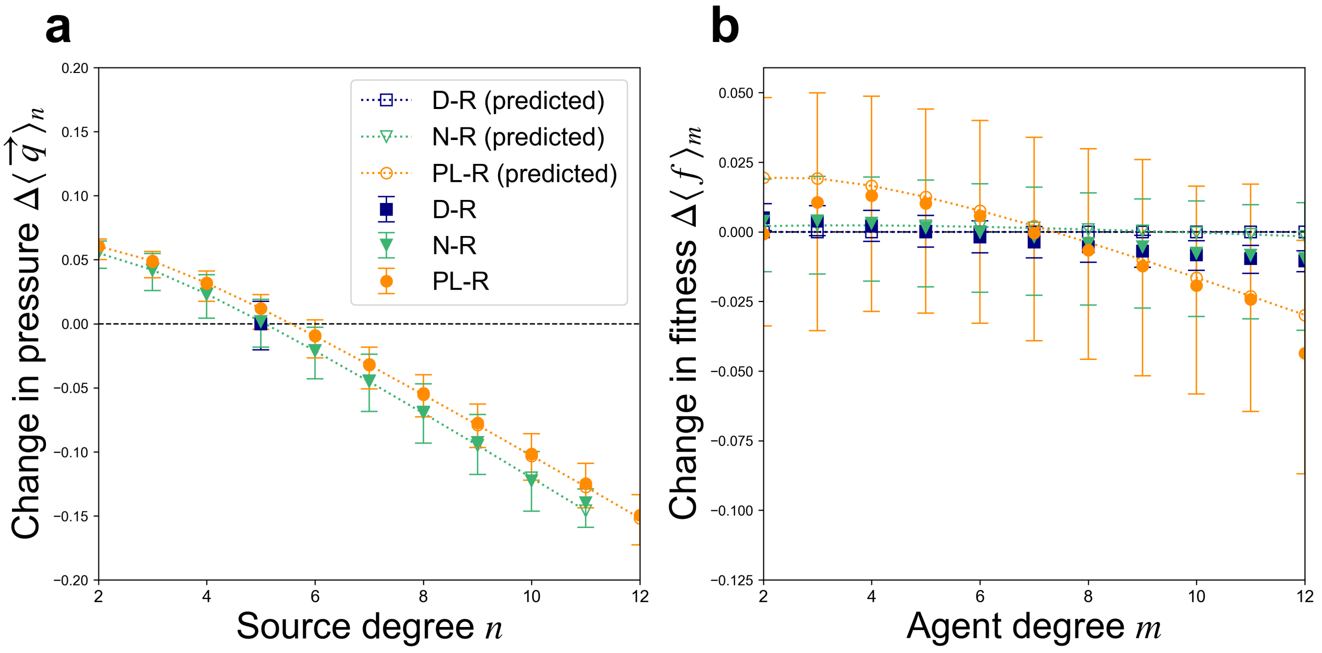

Figure S10. Mean changes in Nash equilibrium extraction patterns under reallocation dynamics with costly extraction (Random agent degree distributions): (a) Change in total extraction pressure $\Delta\langle \overrightarrow{q} \rangle_n = \langle \overrightarrow{q} \rangle_{n,\mathrm{SS}} - \langle \overrightarrow{q} \rangle_{n,\mathrm{Eq}}$, as a function of source degree n; and (b) change in expected agent fitness $\Delta\langle f \rangle_m = \langle f \rangle_{m,\mathrm{SS}} - \langle f \rangle_{m,\mathrm{Eq}}$ as a function of agent degree m for the *degree-proportional capacity* scenario ($\alpha_0 = \beta_0 = 1$, $\gamma_0 = 0.2$). Lower and upper error bars indicate the 2.5th and 97.5th percentiles, respectively.

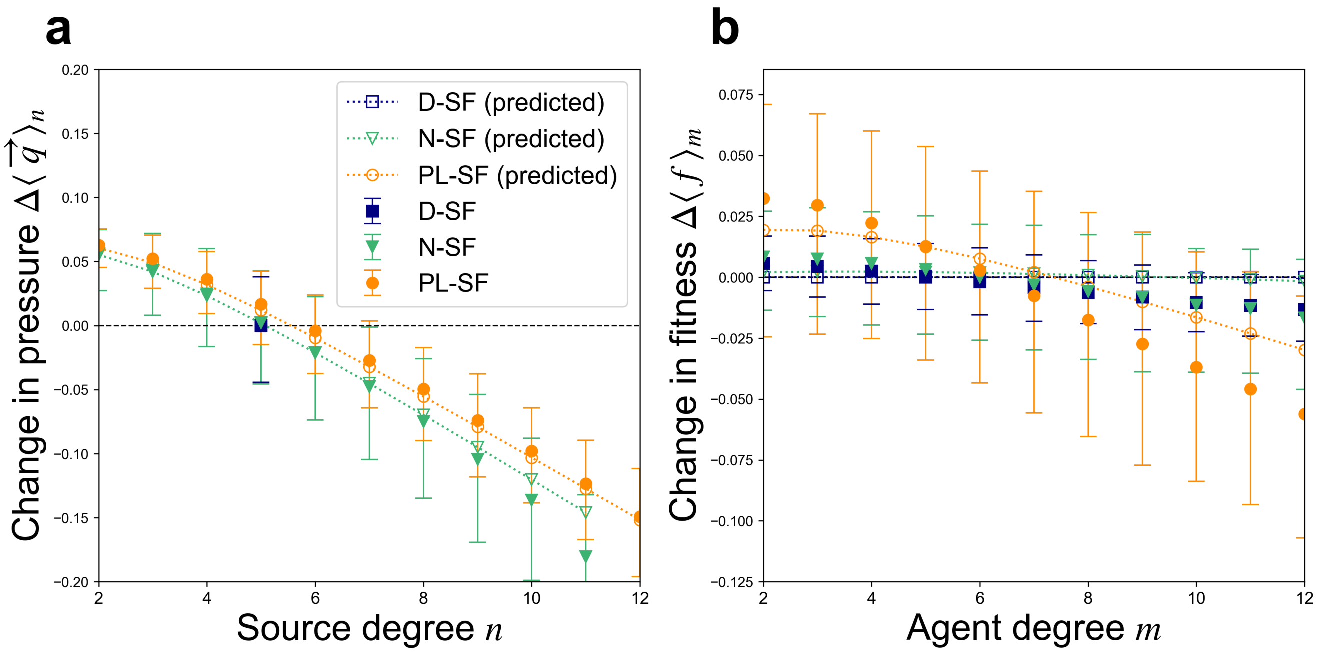

Figure S11. Mean changes in Nash equilibrium extraction patterns under reallocation dynamics with costly extraction (Scale-free-type degree distributions): (a) Change in total extraction pressure $\Delta\langle \overrightarrow{q} \rangle_n = \langle \overrightarrow{q} \rangle_{n,\mathrm{SS}} - \langle \overrightarrow{q} \rangle_{n,\mathrm{Eq}}$, as a function of source degree n; and (b) change in expected agent fitness $\Delta\langle f \rangle_m = \langle f \rangle_{m,\mathrm{SS}} - \langle f \rangle_{m,\mathrm{Eq}}$ as a function of agent degree m for the *degree-proportional capacity* scenario ($\alpha_0 = \beta_0 = 1$, $\gamma_0 = 0.2$). Lower and upper error bars indicate the 2.5th and 97.5th percentiles, respectively.

Network type	Equilibrium efficiency (%)	Equilibrium Gini index	Efficiency increase (%)	Gini index decrease
D-U	67.4	0	0	0
D-R	68.1	.086	0.0	.013
D-SF	71.1	.176	0.2	.027
N-U	66.4	.031	0.5	.027
N-R	67.2	.088	0.5	.015
N-SF	71.1	.187	0.8	.036
PL-U	61.1	.074	3.5	.068
PL-R	61.9	.093	3.4	.045
PL-SF	63.5	.221	4.0	.122

Table S4. Ensemble mean values of equilibrium efficiency $F_{\mathrm{Eq}}/F_{\mathrm{Ef}}$, Gini index G_{Eq}, change in efficiency $(F_{\mathrm{SS}} - F_{\mathrm{Eq}})/F_{\mathrm{Ef}}$, and decrease in Gini index $(G_{\mathrm{SS}} - G_{\mathrm{Eq}})$ for 9 network types. Results correspond to a *degree-proportional capacity* scenario with costly extraction ($\alpha_0 = \beta_0 = 1$, $\gamma_0 = 0.2$).
